# Revealing Robust Atomic Configurations of the Ligand‐Assisted Synthesized High‐Entropy PtIrFeCoNiCu Nano‐Intermetallic Catalysts During Oxygen Reductions in Fuel Cells

**DOI:** 10.1002/advs.202517892

**Published:** 2025-11-18

**Authors:** Yuting Jiang, Qing Zhang, Jing Sun, Cailin Xiao, Tianshou Zhao, Lin Zeng

**Affiliations:** ^1^ Department of Mechanical and Aerospace Engineering The Hong Kong University of Science and Technology Clear Water Bay, Kowloon SAR Hong Kong 999077 China; ^2^ Department of Mechanical and Energy Engineering SUSTech Energy Institute for Carbon Neutrality Southern University of Science and Technology Shenzhen 518055 China

**Keywords:** high‐entropy intermetallics, proton exchange membrane fuel cells, structure‐performance correlation, surface evolution

## Abstract

Despite excellent catalytic performance via the “cocktail effect” and the phase stability of high‐entropy alloys (HEAs), their multicomponent surface remains inadequately explored after oxygen reduction reaction (ORR). Moreover, the facile synthesis of nano HEAs is required for practical applications. Herein, nanosized (∼4.8 nm) carbon‐supported PtIrFeCoNiCu intermetallics (PtIr‐iHEA/C), ∼90% ordered, are prepared through a mercaptosuccinic acid (MSA)‐assisted strategy. Combining atomic‐level characterizations and theoretical calculations, the activity is correlated with the evolving surface configuration. Leveraging the activated Pt and Fe sites, PtIr‐iHEA/C displays an initial mass activity (MA) of 1.65 A mg_Pt/Ir_^−1^ (0.90 V vs RHE) in rotating disk electrodes (6.9 times that of Pt/C), notably with negligible E_1/2_ degradation after 50 000 potential cycles. However, post‐characterizations suggest the cycling induces transition metal (TM) leaching, reconstructing the PtIr‐iHEA@Pt core–shell structure. Theoretical analysis attributes the durable performance tothe electronically optimized rigid Pt shell (active sites) and the strain‐anchored sublayer TMs. Consequently, the fuel cell incorporating PtIr‐iHEA@Pt/C delivers a high mass‐normalized peak power density of 11.6 W mg_Pt/Ir_^−1^ (H_2_/Air), and 79% MA retention from 0.75 A mg_Pt/Ir_^−1^ after cycling (DOE targets: 0.44 A mg_Pt_^−1^, 60% retention). This study uncovers structure‐performance correlations of Pt‐based iHEA for acidic ORR, enlightening the rational HEA design for broader applications.

## Graphical abstract

A facile ligand‐assisted strategy is developed to synthesize nanosized high‐entropy PtIr‐iHEA/C intermetallic catalyst (∼4.8 nm, 90% ordered). Structural analysis during oxygen reduction reveals its surface reconstructions, leading to a core–shell atomic configuration (PtIr‐iHEA@Pt) that originates the superior durability. Combining the high‐entropy constitution, ordered phase, and core–shell structure, the PtIr‐iHEA@Pt demonstrates potential in developing durable and cost‐effective fuel cells.

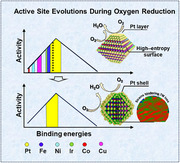

## 1 Introduction

Proton exchange membrane fuel cells (PEMFCs), which offer high‐power density and rapid hydrogen refueling, represent a promising sustainable power source for vehicular applications.^[^ ^1^ ^]^ However, the substantial overpotential caused by the sluggish kinetics of the oxygen reduction reaction (ORR) greatly hinders the performance of PEMFCs.^[^ ^2^ ^]^ Although carbon‐supported Pt nanoparticles (Pt/C) are commercially used as ORR catalysts, the commercialization of PEMFCs remains limited because of their high costs^[^ ^3^, ^4^ ^]^ and the degradation of Pt nanoparticles under dynamic potential conditions.^[^ ^5^, ^6^, ^7^, ^8^ ^]^ Owing to the enhanced performance derived from well‐defined electronic structures, Pt‐based alloys, especially Pt intermetallics, are promising candidates to address these issues.^[^ ^2^, ^9^, ^10^ ^]^

The exceptional structural stability and functional versatility of single‐phase high‐entropy alloys (HEAs), which are derived from their tunable atomic configurations and high‐entropy structures, have recently garnered significant attention in catalysis.^[^ ^11^, ^12^, ^13^, ^14^, ^15^, ^16^ ^]^ Despite the prevalence of face‐centered cubic (*fcc*) solid solutions in HEAs, the ability of specific transition metals (TM, e.g., Fe, Co, Ni, Cu, Zn) with compatible atomic sizes and electronegativities to form ordered intermetallic phases (*P4/mmm*) paves the way for designing Pt‐based high‐entropy intermetallics (Pt‐HEIAs) that demonstrate superior oxygen reduction reaction (ORR) activity and durability.^[^ ^17^, ^18^ ^]^ The “cocktail effect” in catalytic HEAs is known to activate TMs.^[^ ^11^, ^17^, ^19^, ^20^, ^21^, ^22^, ^23^ ^]^ However, their susceptibility to acid leaching remains a significant concern. Consequently, even for phase‐stable Pt‐HEIAs with robust overall durability, the stability of individual TM active sites is questionable, which also poses a risk to PEMFC Nafion membranes through metal dissolution.^[^ ^24^ ^]^ Resolving the evolution of active sites under operation is thus crucial for understanding the ORR behavior of these materials.

The solid‐solution HEA nanoparticles allow for rapid synthesis with random elemental distributions within minutes,^[^ ^25^, ^26^, ^27^ ^]^ whereas the kinetic barriers to atomic ordering impede the fabrication of nanosized Pt‐iHEAs. Their synthesis demands extended annealing at temperatures exceeding 900 °C, which leads to pronounced nanoparticle coarsening through Ostwald ripening and agglomeration.^[^ ^25^, ^26^, ^27^, ^28^ ^]^ Although highly mesoporous carbon materials are typically employed to confine nanosized catalysts and prevent their agglomeration,^[^ ^29^ ^]^ this strategy fails at temperatures near 1000 °C. A robust solution involves using sulfur‐doped mesoporous carbon, which strongly stabilizes metal nanoparticles against sintering via metal–S─C interactions,^[^ ^30^, ^31^ ^]^ suggesting a more efficient route for nanoparticle stabilization.

Herein, we developed a facile route to nanosized (∼4.8 nm) intermetallic PtIr high‐entropy alloys (PtIr‐iHEA/C) by leveraging a cost‐effective impregnation‐pyrolysis process mediated by mercaptosuccinic acid (MSA). The bifunctional MSA molecule concurrently chelates metal precursors during impregnation and suppresses high‐temperature sintering through strong metal‐S─C bonding, ensuring the formation of fine nanoparticles. Density functional theory (DFT) calculations indicate that both noble and non‐noble surface atoms serve as ORR‐active sites, yielding a high initial mass activity of 1.65 A mg_Pt/Ir_^−1^ at 0.90 V (vs RHE) with exceptional durability. However, characterizations after catalyst aging revealed surface reconstruction into a PtIr‐iHEA@Pt core–shell structure, indicating the failure of the surface “cocktail effect.” Nevertheless, the high‐entropy intermetallic core and the robust Pt shell ensure the structural stability of the catalysts, with the electronically refined Pt shell engendering the stable performance in fuel cells.

## 2 Results and Discussion

### 2.1 Synthesis and Characterization

**Figure 1:**
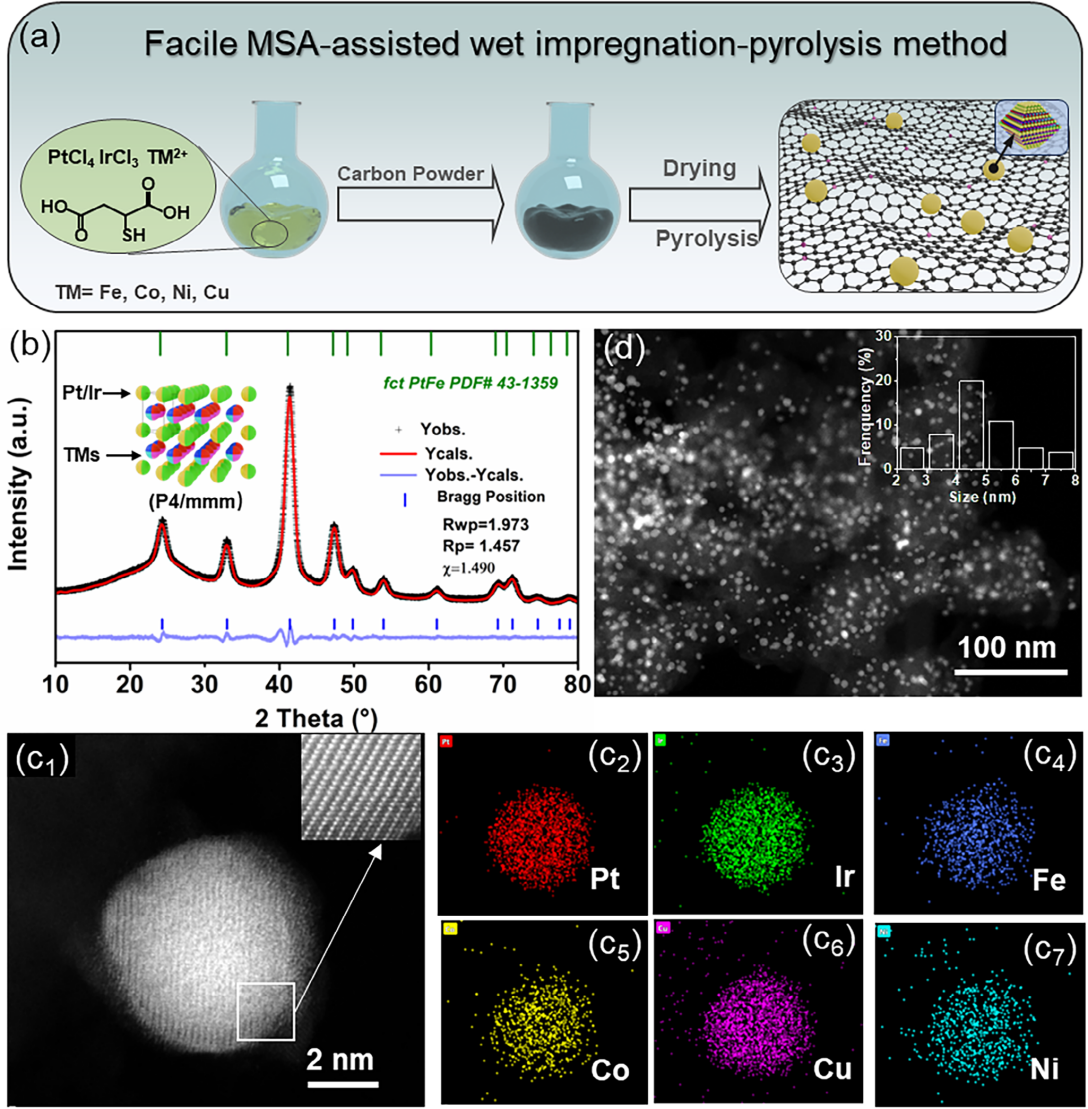
a) Schematic diagram for the preparation of PtIr‐iHEA, b) XRD patterns for PtIr‐iHEA/C, c) HAADF‐STEM image of PtIr‐iHEA and the corresponding element mappings of Pt, Ir, Fe, Co, Ni, and Cu, d) TEM image of PtIr‐iHEA, inset showing the particle distribution.

The synthesis of nanosized PtIr‐iHEA/C (ΔS_mixing_ = 1.58R) employed MSA as a molecular mixer during impregnation‐pyrolysis (**Figure** **1**a). Spectroscopic analysis (Figures S1 and S2, Supporting Information) confirmed that MSA preferentially coordinated Pt and transition metal ions to its ─SH and ─COOH groups, respectively, creating a well‐mixed molecular precursor (Figure S3, Supporting Information). Thermal annealing then provided the necessary activation for atomic diffusion, driving the transition to a long‐range ordered intermetallic structure (Figures S4 and S5, Supporting Information). Following annealing at 1050 °C, the XRD pattern of the as‐prepared PtIr‐iHEA closely matches that of an ordered face‐centered tetragonal (*fct*) PtFe intermetallic phase with an estimated ordering degree of ≈90% (Figure 1b), where slight peak shifts are attributed to differences in atomic sizes among the TMs. The high‐angle annular dark field scanning transmission electron microscopy (HAADF‐STEM) imaging shows well‐defined alternating arrangements of noble and TMs (Figure 1c1). This, combined with the homogeneous spatial distribution of all constituent elements (Pt, Ir, Fe, Co, Ni, Cu, Figures 1c2–c7; S6, Supporting Information), confirms an atomically ordered high‐entropy structure (Table S1, Supporting Information). The catalyst also exhibits uniform nanoparticle dispersion and a narrow size distribution about4.8 nm (Figures 1d; S5, Supporting Information), indicating remarkable thermal stability against sintering, as compared with the unaddition of MSA (Figure S7, Supporting Information). The anti‐sintering stability of the catalyst can be ascribed to the sulfur‐doped carbon support and the formation of few‐layer carbon coatings around the nanoparticles, which collectively enhance metal‐support interactions and inhibit particle agglomeration (Figures S8 and S9, Supporting Information).^[^ ^30^ ^]^

**Figure 2:**
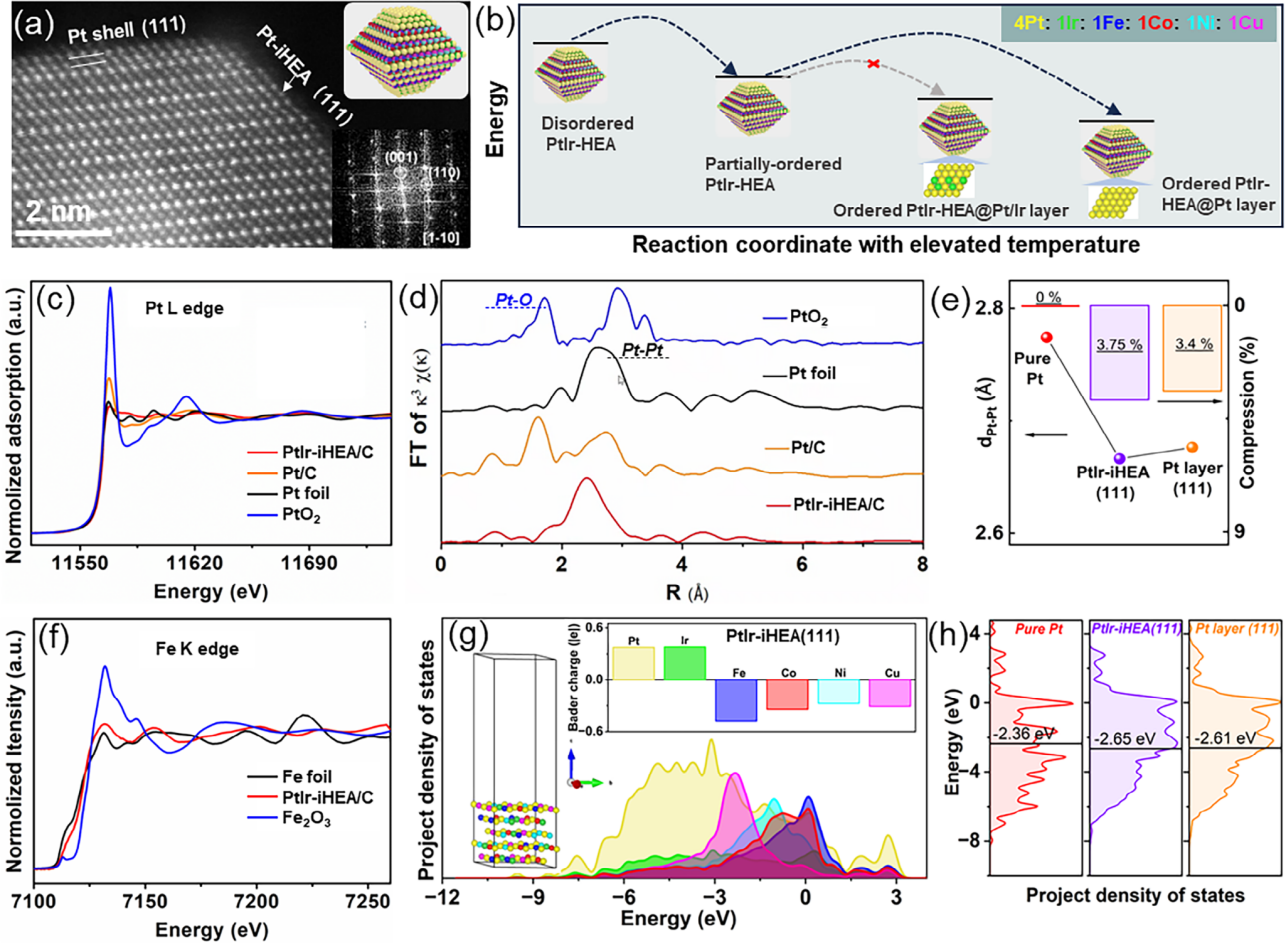
Atomic resolution structure and electronic characterization of PtIr‐iHEA/C. a) HAADF‐TEM image of a single particle after annealing at 1050 °C. Inset is the corresponding FFT‐transformed electronic diffraction patterns, b) Structure evolution of the high entropy alloys at elevated temperature, c,d) The L_3_ edge XANES spectra and the corresponding R space curves of Pt foil, PtIr‐iHEA/C, Pt/C, and PtO_2_. e) Pt–Pt distance with the corresponding compressions on the surfaces of pure Pt, PtIr‐iHEA(111), and Pt layer, simulated by DFT, f) K edge XANES spectra of Fe, g) Project density of states for the simulated PtIr‐iHEA (111) bulk. Inset is the Bader charge on different elements, h) The *d* band center of Pt elements in pure Pt and PtIr‐iHEA(111), and the Pt layer, calculated by DFT (Pt atoms on the top 2 layers are chosen in all samples for the *d‐*band center calculation).

Atomic‐resolution characterization was conducted to elucidate the PtIr‐iHEA/C structure. The image along the [1‐10] zone axis (**Figure** **2**a) shows a well‐ordered intermetallic lattice, matching the simulated *fct* structure (Figure S10, Supporting Information), and unambiguously confirming the *P4/mmm* space group. Notably, a noble‐metal‐terminated (111) facet was consistently observed (Figure S11, Supporting Information), revealing surface segregation, due to more noble elements than TMs (noble:TM = 5:4, nominally). Previous studies have shown that the high surface and cohesive energy of Ir drive it toward the subsurface, stabilizing PtIr‐based alloy structures with Ir‐rich cores and Pt‐terminated surfaces.^[^ ^32^, ^33^ ^]^ Consequently, a pure Pt layer, rather than a mixed Pt/Ir layer, forms at the surface (Figure 2b), which is consistent with the lower surface energy of Pt (Figure S12, Supporting Information). The subsurface Ir atoms further enhance the stability of the outer Pt layer due to their strong acid resistance.^[^ ^33^ ^]^ Moreover, DFT simulations identified the most stable atomic configuration on the exposed PtIr‐iHEA (111) facet (Figure S13, Supporting Information). This unique configuration facilitates direct probation of ORR performance on the Pt (111) overlayer and the ordered PtIr‐iHEA (111) within the same catalyst particle.

X‐ray absorption spectroscopy (XAS) was employed to probe the electronic structure of the PtIr‐iHEA alloy. In the X‐ray absorption near‐edge structure (XANES) spectra, the suppressed white line intensity at both Pt‐L_3_ and Ir‐L_3_ of PtIr‐iHEA indicates enhanced oxidation resistance of Pt and Ir atoms (Figures 2c; S14, Supporting Information).^[^ ^34^ ^]^ Consistent with this, the X‐ray absorption fine structure (EXAFS) spectra show negligible Pt–O contributions in PtIr‐iHEA/C (Figure 2d), suggesting more metallic Pt sites are available.^[^ ^33^, ^35^ ^]^ Moreover, a shorter Pt‐Pt (Ir) distance was obtained compared to that of Pt foil, resulting in compressive‐strain effects on the noble sites (Figure S15 and Table S2, Supporting Information), (strain effect),^[^ ^36^, ^37^ ^]^ quantified as 3.75% and 3.4% on the exposed PtIr‐iHEA (111) and Pt‐layer (111) facets by DFT, respectively (Figure 2e). XANES spectra at the K‐edges of TMs show slight positive shifts relative to the metal foils (Figures 2f; S16, Supporting Information), and no obvious TM─O bond was observed (Figure S17, Supporting Information), implying electron transfers from TMs to Pt(Ir) atoms due to their differing electronegativities (ligand effect),^[^ ^35^ ^]^ which is further confirmed by the extensive overlap of projected density states (PDOS) of different elements with the opposite Barder charges on nobles and TMs (Figure 2g).^[^ ^38^ ^]^ Additionally, the highly compatible electronic state distributions of Pt and Ir suggest the formation of Pt─Ir bonds, benefiting the stability of Pt atoms.^[^ ^33^ ^]^ Originating from the combined geometric and electronic effects, a downshift of ≈0.3 eV in the Pt *d*‐band center is observed for the Pt‐layer (111) and PtIr‐iHEA (111) facets relative to that of pure Pt (Figures 2h; S18, Supporting Information). This electronic modulation optimizes the adsorption strength of oxygenated species, resulting in accelerated ORR rates.^[^ ^39^, ^40^ ^]^

### 2.2 Investigations of Active Surface Configurations on Pristine PtIr‐iHEA/C

**Figure 3:**
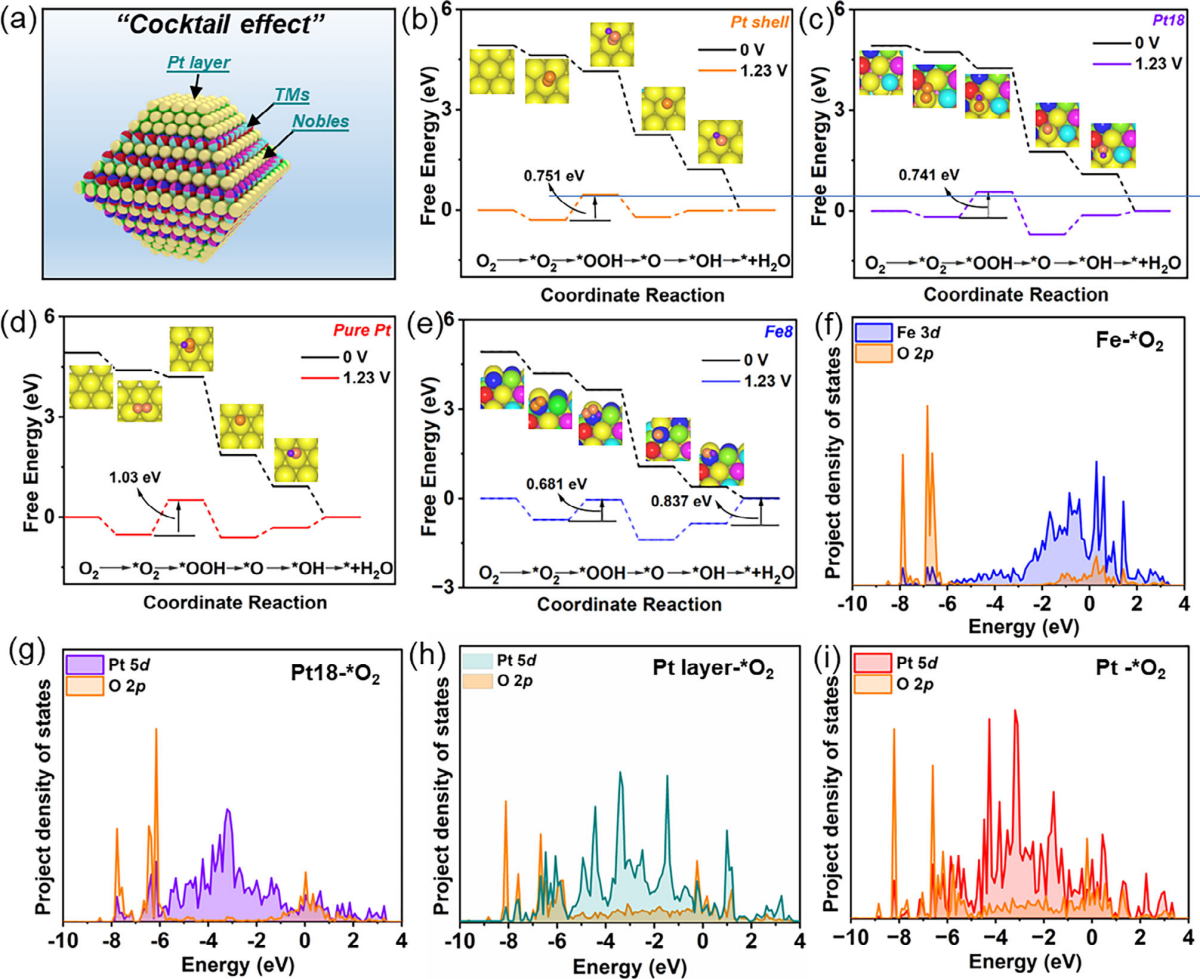
Investigations of the ORR active sites for PtIr‐iHEA nanoparticles. a) Illustration of the exposed potential active sites on PtIr‐iHEA(111) and the segregated Pt layer, b–e) ORR free energy diagrams for Pt‐layer, PtIr‐iHEA(111)‐Pt18, pure Pt, and PtIr‐iHEA(111)‐Fe8, respectively. f–i) Projected density states of active sites‐^*^O_2_ in the chemical adsorption step for Fe8, Pt18, Pt layer, and pure Pt (111), respectively.

PtIr‐iHEA/C leverages its multi‐element composition to create a variety of potential active sites, including exposed Pt‐layer and PtIr‐iHEA (111) (**Figure** **3**a), which were subsequently evaluated using DFT simulations for ORR catalysis. On unmodified Pt, strong binding to intermediates imposes a large activation barrier for the electron–proton transfer step ^*^O_2_ + H⁺ + e^−^  → ^*^OOH, thereby limiting ORR efficiency.^[^ ^35^, ^41^ ^]^ The ^*^OOH formation barrier is markedly lowered on the Pt‐layer (0.751 eV) and PtIr‐iHEA (111) (0.741 eV) compared to pure Pt (1.03 eV)‐a consequence of their downshifted *d*‐band centers (Figure 2h) that optimize adsorption and facilitate the EPT process.^[^ ^10^, ^42^ ^]^ Moreover, the Ir8 site on the PtIr‐iHEA (111) facet exhibits a similar energy barrier of 1.06 eV to pure Pt (Figure S19, Supporting Information), indicating its function as an active site. The ΔG_*O_‐ΔG_*OH_ values of non‐noble metals are between 0.5 and 1.0 eV, generating potential ORR activity,^[^ ^43^ ^]^ which suggests Fe8 on PtIr‐iHEA (111) can most promisingly catalyze the ORR process with a ΔG_*O_‐ΔG_*OH_ of 0.716 eV (Figures S20 and S21, Supporting Information). As a result, Fe8 delivers a rate‐determining energy barrier of 0.837 eV (Figure 3e), significantly lower than that of pure Pt (1.030 eV), indicating the high activity of Fe8 sites. Intrinsically, the smaller orbital overlaps between the active sites (Fe8/ Pt18/ Pt layer) and O_2_ (Figure 3f–h), compared to pure Pt‐^*^O_2_ (Figure 3i) indicate weaker O_2_ adsorption on Fe8, Pt18, and Pt layer. The weakened O_2_ adsorption can facilitate the subsequent EPT step, significantly reducing the ^*^OOH formation energy barrier to 0.681 eV (Fe8), 0.741 eV (Pt18), and 0.751 eV (Pt layer) (Pt:1.030 eV).^[^ ^17^ ^]^ Moreover, the rapid reduction of adsorbed O_2_ decreases its coverage on active sites, thereby liberating more sites for ORR. Thus, the exposed multi‐element composition introduces new ORR active sites,^[^ ^11^ ^]^ prospectively enhancing the ORR performance of the PtIr‐iHEA catalyst.

### 2.3 ORR Performance of PtIr‐iHEA/C by the RDE Technique

**Figure 4:**
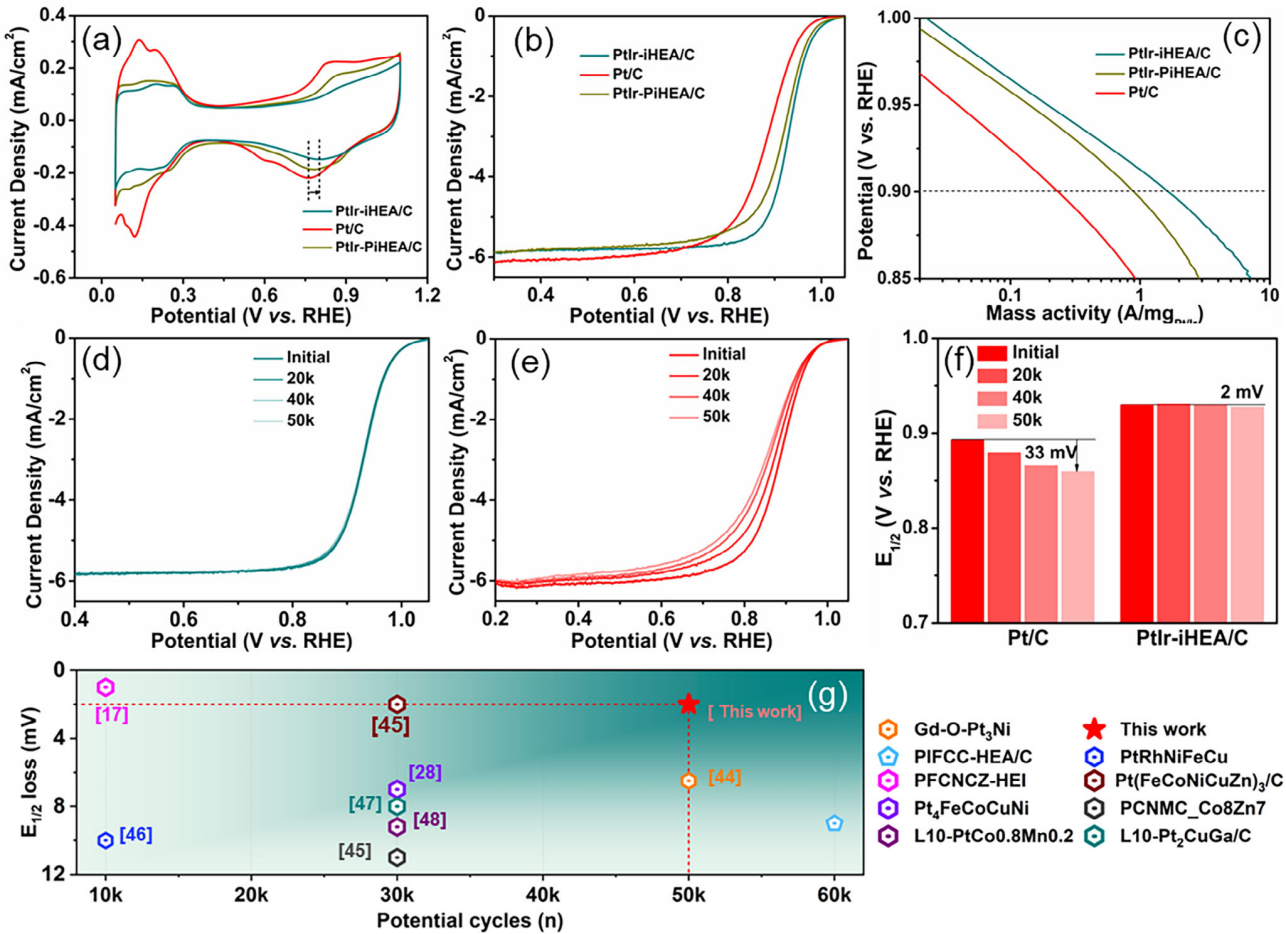
Electrochemical characterization of the catalysts. a–c) CV, LSV, and mass activity curves of Pt/C, PtIr‐iHEA/C, and PtIr‐PiHEA/C, d,e) LSV curves for Pt‐iHEA and Pt/C during durability test, respectively, f) Half potential changes of PtIr‐iHEA/C and Pt/C during durability test, g) Stability comparisons of the best reported Pt alloys.^[^ ^17^, ^18^, ^28^, ^42^, ^44^, ^45^, ^46^, ^47^, ^48^ ^]^

The ORR performance of the catalyst was evaluated in 0.1 M HClO_4_. The electrochemical surface area (ECSA) of PtIr‐iHEA/C, determined from the Hupd region (hydrogen desorption peak), was 36.7 m^2^ g_Pt/Ir_^−1^, which is lower than that of Pt/C (66.5 m^2^ g_Pt_^−1^) and the partially ordered PtIr‐PiHEA/C (40.2 m^2^ g_Pt_^−1^) (**Figure** **4**a). This trend is consistent with the relatively larger nanoparticle size of the PtIr‐iHEA/C catalyst (Figure S5a_2_–c_2_, Supporting Information). The reduction potential peak of PtO_x_ is critical for evaluating the ORR activity of Pt‐based catalysts, as it reflects the competition between ORR and Pt oxidation.^[^ ^34^ ^]^ A positively shifted PtO_x_ reduction potential in PtIr‐iHEA (Figure 4a) signifies enhanced anti‐oxidation capability (Figure 4a), where the synergistic strain and ligand effects yield a kinetically optimized, weakened oxophilicity that thereby accelerates the ORR.^[^ ^35^, ^49^, ^50^ ^]^ Furthermore, DFT simulations suggest that Fe8 and Ir8 sites on PtIr‐iHEA(111) also contribute to the ORR activity (Figure 3c–e). As a result, the LSV curves of the alloys are positively shifted compared to that of Pt/C (Figure 4b), with PtIr‐iHEA/C and PtIr‐PiHEA/C showing MA of 1.65 and 0.86 A mg_Pt/Ir_^−1^ at 0.90 V, 6.9 and 3.7 times that of Pt/C (0.24 A mg_Pt_^−1^) (Figure 4c). The superior kinetics and specific activity (SA) of PtIr‐iHEA/C are corroborated by its lower Tafel slope and enhanced SA (Figures S22 and S23, Supporting Information), collectively demonstrating the exceptional activity of the high‐entropy alloys. Moreover, the activity trend of PtIr‐PiHEA/C and PtIr‐iHEA/C underscores a clear structure‐activity relationship, where higher atomic ordering leads to superior ORR performance.^[^ ^51^ ^]^

Accelerated stress tests (AST) in an O_2_‐saturated environment reveal the superior stability of PtIr‐iHEA/C, which shows a negligible E_1/2_ loss of 2 mV after 50 000 cycles (Figure 4d,f). Whereas, under identical conditions, Pt/C undergoes significant degradation, with an E_1/2_ loss of 33 mV (Figure 4e,f). After AST, PtIr‐iHEA/C maintains a high mass activity of 1.44 A mg_Pt/Ir_^−1^ at 0.90 V, far surpassing Pt/C (0.12 A mg_Pt_^−1^, Figure S24, Supporting Information). The severe activity degradation of Pt/C is attributed to a 43% loss in ECSA (down to 35.2 m^2^ g_Pt_^−1^, Figure S25a,c, Supporting Information), resulting from nanoparticle agglomeration and dissolution/redeposition under potential cycling (Figure S26a,b, Supporting Information). In contrast, PtIr‐iHEA/C maintains its original ECSA (36.1 m^2^ g_Pt_^−1^, Figure S25b,c, Supporting Information) and nanoparticle dispersion (Figure S27, Supporting Information), demonstrating exceptional structural and anti‐oxidative stability. This robustness, enabled by strong metal‐support interactions,^[^ ^52^ ^]^ positions the catalyst among the most durable ORR catalysts (Figure 4g).

### 2.4 Investigations of Aged PtIr‐iHEA/C: Durable Structure and Active Surface Configurations

**Figure 5:**
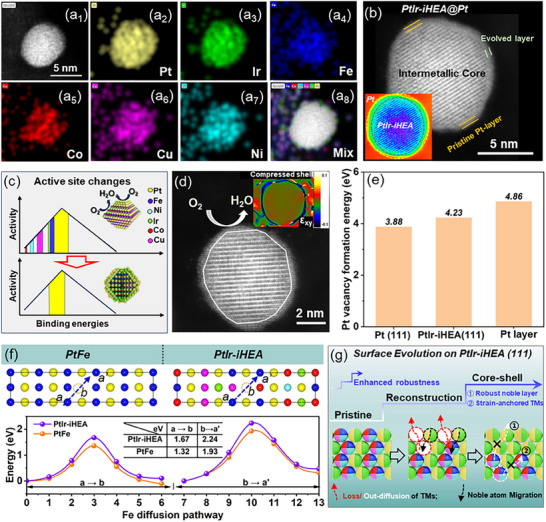
Durability mechanisms of PtIr‐iHEA/C catalyst. a_1_–a_8_) HAADF‐STEM image and the corresponding element mappings of PtIr‐iHEA/C after 50 000 cycles b). HAADF‐STEM image of PtIr‐iHEA/C after AST with the inset of the corresponding core–shell display, c). Schematic of catalytic sites for the PtIr‐iHEA/C catalyst before and after AST, d). Atomic resolution HAADF‐STEM images of PtIr‐iHEA/C after AST with its strain measurement (inset), e). Pt vacancy formation energy for pure Pt, Pt on PtIr‐iHEA (111), and Pt shells, f) Diffusion energy barriers of Fe in binary intermetallic PtFe and the high‐entropy intermetallic PtIr‐iHEA, g). Proposed surface evolution of PtIr‐iHEA/C (111) over potential cycles.

A fundamental aspect of designing durable ORR catalysts involves tracking the evolution of active sites under reaction conditions. However, for structurally stable high‐entropy alloys, such studies are notably scarce, leaving a gap in understanding their true structure‐activity‐durability relationships.^[^ ^13^, ^14^ ^]^ To elucidate the origin of the durable activity, we analyzed the PtIr‐iHEA/C catalyst after AST. Although the elemental composition remains largely preserved (Figure **5**a1–a8), indicating robust structural integrity,^[^ ^13^, ^17^ ^]^ atomic‐resolution imaging reveals surface reconstruction, forming a structure with an intermetallic core enclosed by a noble‐metal shell (PtIr‐iHEA@Pt/C, Figure 5b). The surface evolution analysis confirms that the durable ORR activity stems from the persistent Pt shell (layer) that is electronically modulated by the strained intermetallic core (Figures 2g,h and 5c,d)‐rather than the original PtIr‐iHEA(111) facet with etching of surface TMs. Thus, although transition metal (TM) sites in HEAs are often reported to possess anti‐dissolution characteristics,^[^ ^17^, ^18^, ^53^ ^]^ their inherent instability in acidic ORR conditions limits practical utility. Moreover, minimal change in the ECSA of PtIr‐iHEA (H_upd,_ an indicator for exposed Pt sites) (Figure S25, Supporting Information), implies that the transformation of PtIr‐iHEA(111) into a Pt surface likely completed during the activation stage (Figure S28, Supporting Information).

DFT simulations were performed to assess the structural stability of the cycled PtIr‐iHEA@Pt/C.The calculated Pt vacancy formation energy increases in the order: pure Pt (111) <PtIr‐iHEA (111) <Pt layer (shell) (Figure 5e), suggesting exceptional stability of the Pt layer, which is attributed to the strong alloying effects originating from the ordered intermetallic core.^[^ ^35^ ^]^ Notably, the stability of Pt atoms in PtIr‐iHEA(111) is closely linked to adjacent TMs, suggesting that TM leaching would induce surface reconstruction toward a more stable Pt shell. Concurrently, anisotropic strain within the high‐entropy ordered crystal domains (Figure S29, Supporting Information) retards TM diffusion, as evidenced by a higher activation energy compared to binary intermetallics (Figure 5f). This effectively suppresses TM out‐diffusion and corrosion (Figure 5g), thereby stabilizing the core–shell architecture along with the robust Pt shell.

### 2.5 Fuel Cell Performance of PtIr‐iHEA@Pt/C

**Figure 6:**
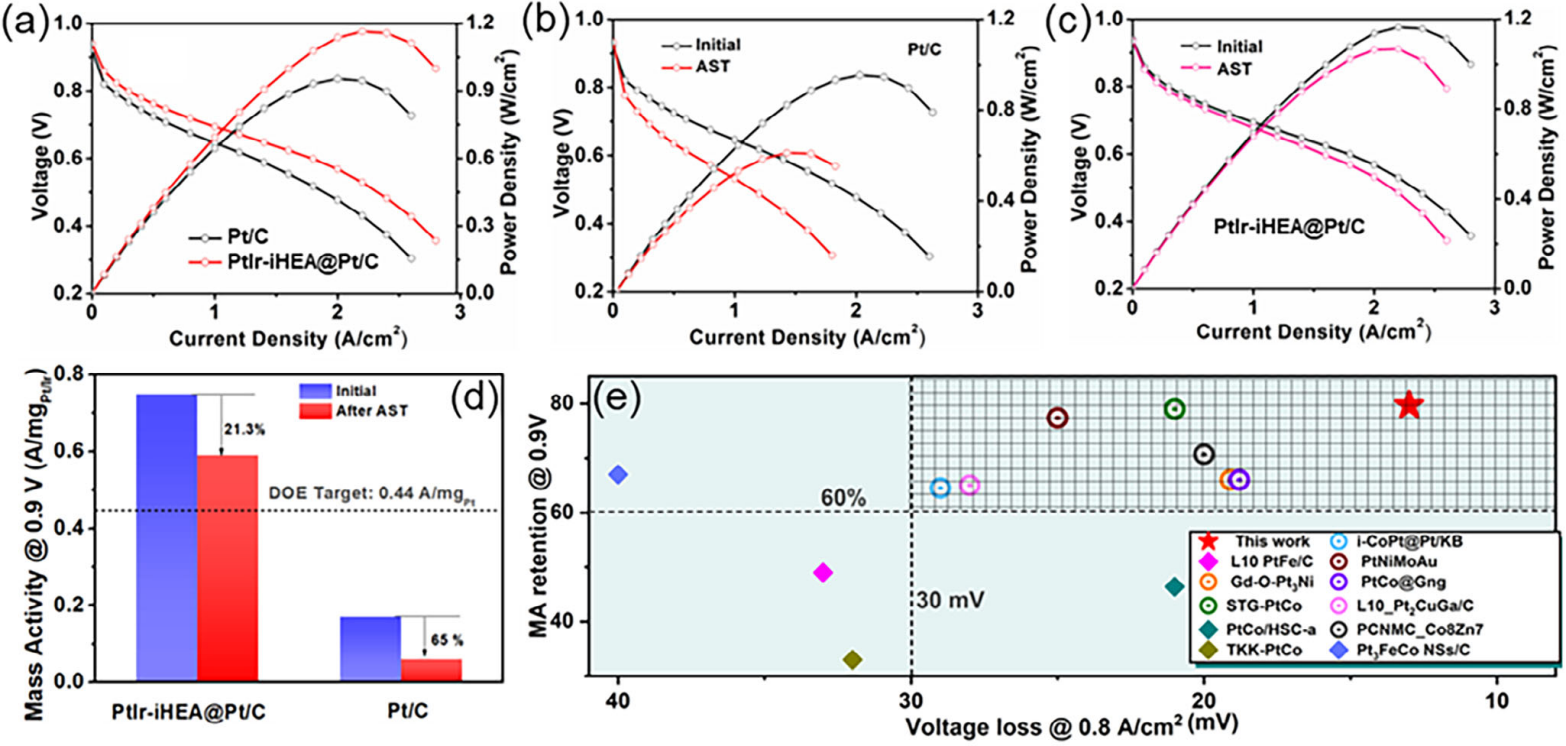
MEA performance of Pt/C and the PtIr‐iHEA@Pt/C catalyst. a). Polarization curves of Pt/C and Pt‐iHEA/C, b,c) Polarization curves of Pt/C and PtIr‐iHEA@Pt/C /C catalysts before and after AST, d) MA changes of Pt/C and PtIr‐iHEA@Pt/C /C catalysts before and after AST, e) Comparations with the best‐reported catalysts in MA retention at 0.90 V and the voltage loss at 0.8 A cm^−2^ after AST (The corresponding references are listed in Table S3 (Supporting Information) along with their peak power density and mass normolizaed power density, Supporting Information).

To eliminate unstable surface TM sites, the PtIr‐iHEA/C catalyst was subjected to acid pre‐leaching (70 °C, 12 h), yielding the PtIr‐iHEA@Pt/C structure (Figure S30, Supporting Information). In membrane electrode assembly (MEA) tests, the pre‐treated catalyst exhibits substantially enhanced fuel cell performance compared to Pt/C (**Figures** **6**a; S32, Supporting Information). PtIr‐iHEA@Pt/C delivers a mass activity of 0.75 A mg_Pt/Ir_^−1^ at 0.90 V, exceeding the DOE 2025 target (0.44 A mg_Pt_^−1^) and surpassing Pt/C (0.18 A mg_Pt_^−1^) by a factor of 4.2. It also achieves a mass‐normalized peak power density of 11.6 W mg_Pt/Ir_^−1^, outperforming Pt/C (9.5 W mg_Pt_^−1^). This enhanced MEA performance is attributed to its high intrinsic ORR activity, stemming from an optimized Pt electronic structure and finely tuned intermediate adsorption.^[^ ^54^, ^55^ ^]^ Durability of the MEAs was assessed following DOE protocols, with markedly different degradation behaviors for Pt/C and PtIr‐iHEA@Pt/C. The Pt/C MEA lost 65% of its initial mass activity with a ∼100 mV voltage decay at 0.8 A cm^−2^, due to nanoparticle coalescence and Ostwald ripening (Figures 6b–d; S31a and S32, Supporting Information). Conversely, the PtIr‐iHEA@Pt/C MEA exhibited superior stability, retaining 78.7% of its MA (0.59 A mg_Pt/Ir_^−1^) with only 13 mV voltage loss, meeting the DOE durability targets, and confirming the structural robustness of the high‐entropy intermetallic catalyst (Figures 6c,d; S31b and S32, Supporting Information). The retention of superlattice peaks after AST (Figure S33, Supporting Information) further corroborates the exceptional structural durability of PtIr‐iHEA@Pt/C, validating its position among the leading ORR catalysts in MEA benchmarks (Figure 5e; Table S3, Supporting Information) and its practical potential for PEMFCs.

## 3 Conclusion

In summary, we report the synthesis of a sub −5 nm high‐entropy intermetallic PtIr‐iHEA/C with ∼90% ordering, achieved through a facile MSA‐mediated impregnation‐pyrolysis strategy. The catalyst presents compressed Pt layers and multi‐component PtIr‐iHEA(111) facets as active planes, yielding a high initial MA of 1.65 A mg_Pt/Ir_^−1^ at 0.90 V (vs RHE). However, the catalyst's durability arises from the in‐situ formation of a compressed core–shell architecture (PtIr‐iHEA@Pt), triggered by the leaching of TMs from the initial PtIr‐iHEA(111) facets, which indicates failure of the “cocktail effect”. When implemented in PEMFCs, the PtIr‐iHEA@Pt/C MEA achieves a mass activity of 0.75  A mg_Pt/Ir_^−1^ at 0.90 V and exceptional durability (13 mV loss at 0.8 A cm^−2^), positioning it among the leading reported ORR catalysts. This work establishes a scalable route for synthesizing ordered high‐entropy nanoalloys and provides fundamental insights into their ORR behavior, advancing the rational design of highly active and stable catalysts for energy conversion.

## Conflict of Interest

The authors declare no conflict of interest.

## Supporting information



Supporting Information

## Data Availability

The data that support the findings of this study are available from the corresponding author upon reasonable request.
