# Supplementary material for: Revealing Robust Atomic Configurations of the Ligand‐Assisted Synthesized High‐Entropy PtIrFeCoNiCu Nano‐Intermetallic Catalysts During Oxygen Reductions in Fuel Cells
Source: Adv Sci (Weinh). 2025 Nov 18;13(7):e17892. doi: 10.1002/advs.202517892 (PMC12866707; doi:10.1002/advs.202517892)
Supplement: Supplementary file 1 — Supporting Information [file ADVS-13-e17892-s001.docx]

**Supporting Information**

**Revealing Robust Atomic Configurations of the Ligand-Assisted Synthesized High-Entropy Nano-Intermetallic Catalysts for Oxygen Reductions in Fuel Cells**

*Yuting Jiang, Qing Zhang,* *Jing Sun, Cailin Xiao, Tianshou Zhao*, and Lin Zeng**

*Yuting Jiang, Qing Zhang, Jing Sun, Cailin Xiao, Tianshou Zhao*, and Lin Zeng**

Y. Jiang, Jing Sun, T. Zhao

Department of Mechanical and Aerospace Engineering,

The Hong Kong University of Science and Technology, Clear Water Bay, Kowloon SAR 999077, China.

E-mail: metzhao@ust.hk

Y. Jiang, Q. Zhang, C. Xiao, T. Zhao, L. Zeng

Department of Mechanical and Energy Engineering

SUSTech Energy Institute for Carbon Neutrality

Southern University of Science and Technology, Shenzhen 518055, China

E-mail: zengl3@sustech.edu.cn

**Table of Contents**

1. Experimental section

2. Results and discussion

3. Reference

**1 Experimental section**

**1.1 Material synthesis**

**Synthesis of highly ordered PtIr-iHEA/C**

The metal salts of PtCl_4_, IrCl_3_, Co(NO_3_)_2_.6H_2_O, Fe(NO_3_)_3_.9H_2_O, Ni(NO_3_)_2_.6H_2_O and Cu(NO_3_)_2_.6H_2_O at a mole ratio of 4:1:1:1:1:1 were dissolved in DI water. Then, the thiomalic acid was added to the above solution with a mole ratio of 2:1 versus PtCl_4_. The solution was kept still for 2 hours before being added to the dispersion of carbon support (EC600) in ethanol. The mixture was then stirred overnight, and the solvent was removed by rotary evaporation. After being dried in an oven at 70 °C for 6 hours, the obtained black powder was annealed in the tube furnace under 10% H_2_/Ar at 1050 °C for 2 hours and maintained at 650 °C for 5 hours. The as-prepared catalysts were treated in air at 220 °C for 2 hours to remove the carbon shells on the surface of the catalysts, followed by annealing at 700 °C (H_2_/Ar) for another 2 hours.

**Synthesis of partially ordered PtIr-iHEA/C (PtIr-PiHEA/C)**

The procedures are the same as those of highly ordered PtIr-iHEA/C except that the annealing temperature was set at 850 °C for 2 hours.

**Synthesis of partially ordered PtIr-iHEA@Pt/C**

The PtIr-iHEA@Pt/C catalyst was obtained by acid etching of PtIr-iHEAt/C in 0.5 M H_2_SO_4_ at 70 °C for 12 hours, followed by throughout washing, and drying at 80°C overnight.

**1.2 Physical characterization**

X-ray diffractometry measurement (XRD) was used to determine the crystal phase of all catalysts on an X-ray diffractometer (SmartLab 3 kW) with Cu target (Kα1=1.5406 Å). The morphologies and element distributions for all catalysts were characterized by transmission electron microscopy (TEM) measurements and scanning transmission electron microscopy (STEM) with energy-dispersive spectroscopy (EDS) on the transmission electron microscope (Talos F200 G2). High-angle annular dark-field scanning transmission electron microscopy (HAADF-STEM, Titan Themis G2) was conducted to visualize the ordered atom arrangement. X-ray photoelectron spectroscopy (XPS, PHI 5000 Versaprobe III) measurements were used to characterize the valence states of elements in different catalysts. The mass loadings of all catalysts were determined by inductively coupled plasma mass spectroscopy (ICP-MS, 7700X) analyses, while the UV absorption measurement was done on a UV-VIS apparatus (UV-3600Plus). The X-ray absorption near-edge structure (XANES) measurements were conducted in Taiwan Photon Source (TPS) to reveal information about the structural and valence information of atoms in different catalysts.

**Ordering degree calculation (based on the XRD patterns):**

$$Ordering degree=\frac{\frac{S_{110}}{(S_{111}+S_{200}+S_{002})}}{\frac{I_{110}}{(I_{111}+I_{200}+I_{002})}}$$

Where S is the integrated areas under (110), (111), (200), and (002) planes for samples; I is the peak intensities of (110), (111), (200), and (002) planes for standard intermetallic *fct* PtFe with the intensity ratio of 0.18667 for (110) versus (111), (200) and (002).

**1.3 Density functional theory (DFT) computation**

All the calculations are performed in the framework of the density functional theory with the projector augmented plane-wave method, as implemented in the Vienna ab initio simulation package.^1^ The generalized gradient approximation proposed by Perdew-Burke-Ernzerhof (PBE) is selected for the exchange-correlation potential.^2^ The cut-off energy for the plane wave is set to 500 eV. The energy criterion is set to 10−5 eV in the iterative solution of the Kohn-Sham equation. All the structures are relaxed until the residual forces on the atoms have declined to less than 0.02 eV/Å. To avoid interlaminar interactions, a vacuum spacing of 20 Å is applied perpendicular to the slab. The Gibbs free energy of the intermediate at 298.15 K and 1 atm was calculated by *G* = *E*abs +ZPE – *TS*, where *E*abs, *ZPE*, and *S* are the adsorption energy with respect to H_2_O and H_2_, zero-point energy, and entropy of the adsorption of intermediates, respectively.^3, 4^ (Table S4)The vacancy formation energies (*E*_vac_) of the atoms were calculated by *E*_vac_ = *E*_vac-M_ + *E*_M_ − *E*_slab_, where *E*_vac-M_ and *E*_slab_ are the total energies of a surface slab with a metal atom vacancy and a vacancy-free surface slab, respectively. *E*_M_ is the energy of a single metal atom in a bulk M crystal.

**1.4 Electrochemical characterization**

**Rotating disk electrode (RDE) measurement**

**Ink preparation**: 2 mg catalyst was dispersed in 1.99 ml isopropanol before the addition of 10 ul Nafion solution (5 wt%), followed by the homogenization step in an ultrasonic machine (ice bath) for 1 hr. The ink was then drop-coated onto the polished glass carbon electrode (GCE) and dried at ambient conditions.

**Cyclic Voltammetry (CV) and linear sweep voltammetry (LSV) measurements**: the electrochemical measurement was conducted in a three-electrode electrolytic cell with the Pt net as the counter electrode, a homemade reversible hydrogen electrode (RHE) as the reference electrode, catalyst-coated GDE as the working electrode, and 0.1 M HClO_4_ as the electrolyte. After bubbling the electrolyte with N_2_ for 0.5 hour, the catalysts were activated by the voltage sweep between 0.05 V and 1.2 V for 20 cycles. Then, the CV curve was recorded between 0.05 V and 1.1 V, and the LSV measurement under N_2_ was also tested from 1.05 V to 0.05 V. Then, oxygen was bubbled into the electrolyte for 0.5 hour. The LSV curve was measured from 1.05 V to 0.05 V at 1600 rmp, and the electrochemical impedance spectroscopy (EIS) was also obtained under the open potential from 0.1 HZ to 100000 HZ to get the electrolyte resistance. The durability test was conducted by CV scanning between 0.6 V and 1.0 V for different cycles (20000, 40000, 50000) in O_2_-saturated electrolyte, with the corresponding LSV curves recorded.

**1.5 Fuel cell test**

**Membrane electrode assembly (MEA) fabrication**: the catalyst-coated membrane (CCM) was fabricated as follows:

Before coating, the ink was prepared at an I/C ratio of 0.85 and homogenized for 1 hr. Then, the noble metal loadings at both the anode and cathode are controlled at 0.1 mg/cm^2^ during spraying coating, with the commercial Pt/C (JM 3000) as the anode catalyst and the high-entropy alloy as the cathode catalyst. Moreover, an MEA with 0.1 mg_Pt_/cm² on both sides (commercial Pt/C) was fabricated to benchmark the alloy MEA. The proton exchange membrane (Gore, 15 μm) was used in all MEAs with an active area of 3.24 cm^2^.

**MEA characterization**

The CCM was sandwiched between two sealing PTFE strips (150 μm) followed by the gas diffusion layer (GDL, 230 μm), before being compacted by the graphite plate with flow fields and the Au-coated current collectors. The cell was activated by voltage sweeping from the open voltage to 0.4 V until the membrane was fully humidified and a stable polarization curve was obtained (80 °C; 100% RH; 0.5 lsmp H_2_; 1.0 lsmp air). Then, the cell temperature was set to 40 °C and kept at 0.3 V for 6 hours under 150 % RH, during which the generated liquid water is expected to remove the adsorbed impurities on the catalysts. After the activation process, the polarization curve was recorded by current sweeping (200 kPa; 80 °C; 100 % RH; 0.5 lsmp H_2_; 1.0 lsmp air or oxygen). For the durability test, potential sweeping between 0.6 V and 0.95 V was cycled for 30000 times with durations of 3 s at both potentials each cycle (80 °C, 100 % RH 0.1 lsmp H_2_, anode / 0.05 lsmp N_2_, cathode).

**2. Results and discussion**

**Complexing behavior between MSA and metal ions:**


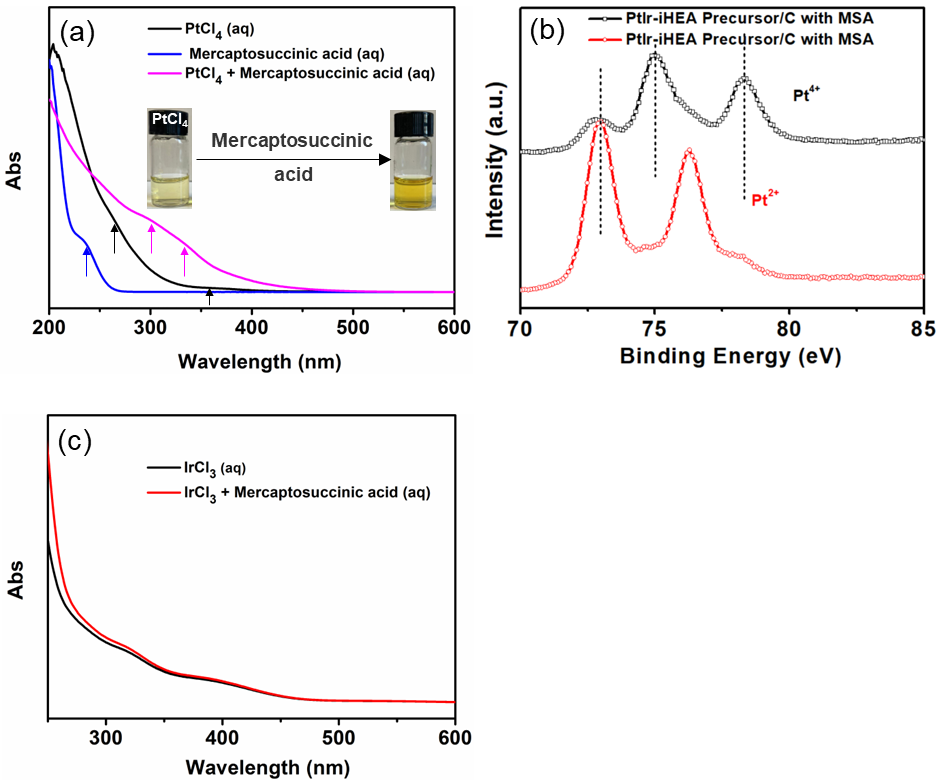


**Figure S1** (a) UV–Vis spectra of the PtCl_4_ solution, MSA acid, and PtCl_4_+MSA solution; (b) XPS spectrum of Pt-MSA complex; (c) UV–Vis spectra of the IrCl_3_ solution and IrCl_3_+MSA solution


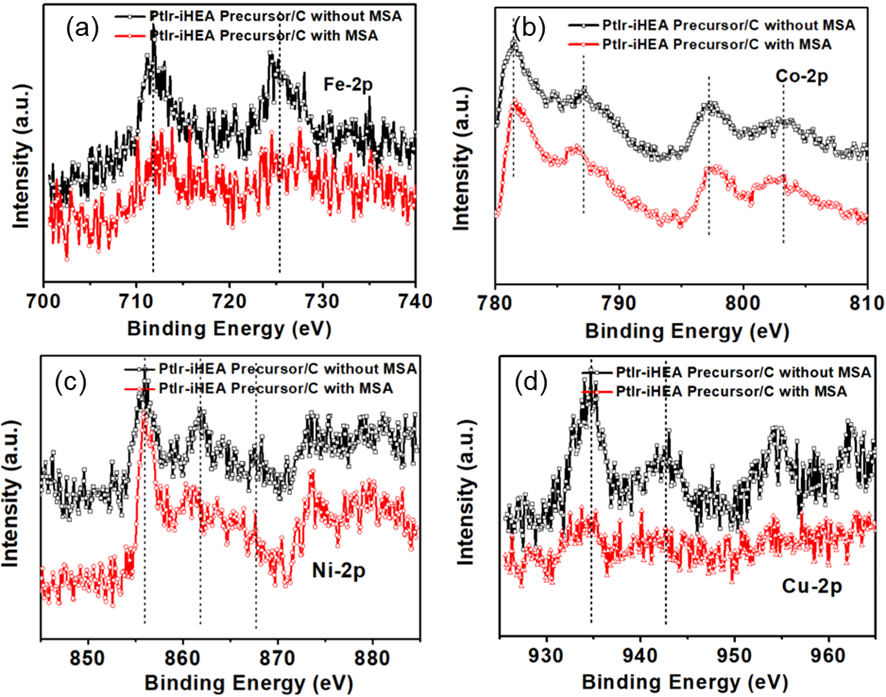


**Figure S2.** (a-d) XPS spectra of Fe, Co, Ni, and Cu in the PtIr-iHEA precursor with and without MSA, respectively.

The interactions between MSA and metal ions were investigated. UV-Vis spectra reveal distinct peaks for the PtCl_4_ + MSA solution compared to the pristine PtCl_4_, indicating coordination evolution of the Pt centers, further supported by the color change of the PtCl_4_ solution from light yellow to darker yellow (**Figure S1a**). Additionally, X-ray photoelectron spectroscopy (XPS) spectra of Pt 4f (**Figure S1b**) show reduced Pt valence states with red-shifted peaks in the Pt-based complex, resembling Pt(II) ions coordinated by sulfur-containing ligands, indicating that Pt atoms are coordinated to sulfur atoms in the Pt-MSA complex.^5, 6^ In contrast, negligible changes were observed in the UV-Vis spectra of the IrCl_3_ solution after mixing with MSA ligands (**Figure S1c**), indicating weak interactions between Ir(III) ions and MSAs. XPS peaks for the transition metal ions also exhibit shifts in the PtIr-iHEA precursor/MSA mixtures (**Figure S2**), suggesting the possibility of transition metals and Pt ions forming a single complex through the three coordinating sites of MSAs, as shown in **Figure S3**, which facilitates the mixing of elements before pyrolysis.


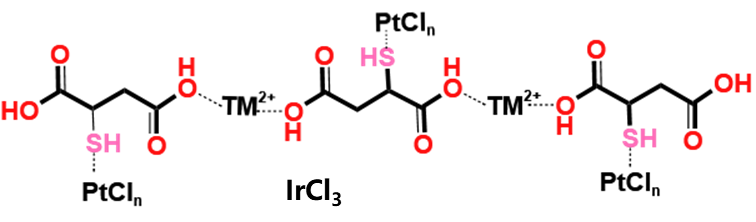


**Figure S3.** Possible atomic configurations in the metal ion-MSA complex


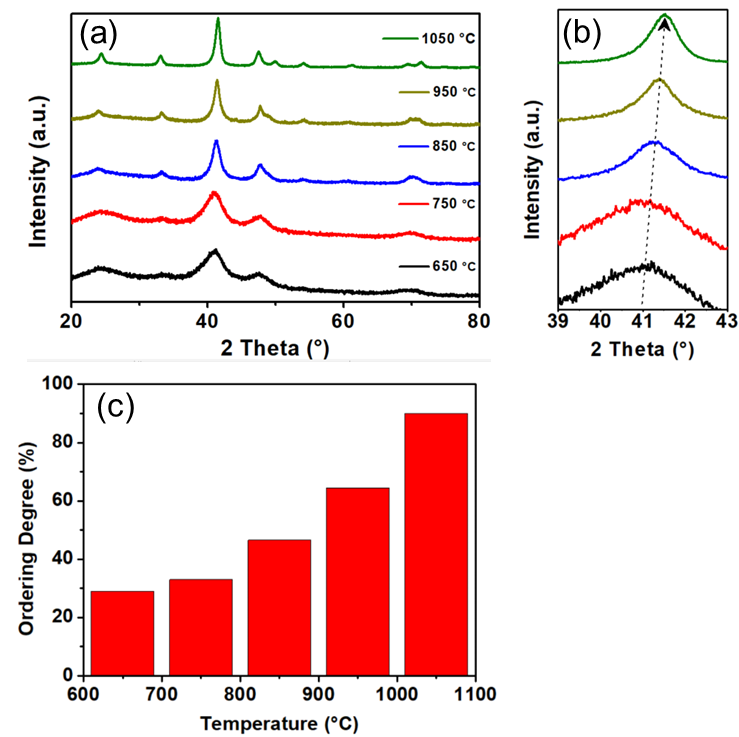
**Phase and morphology evolution**

**Figure S4.** (a) ex-situ XRD patterns of PtIr-HEA obtained under different temperatures; (b) Enlarged (111) diffraction peaks of the alloys; (c) Ordering evolution of the high-entropy intermetallic upon increasing temperature.


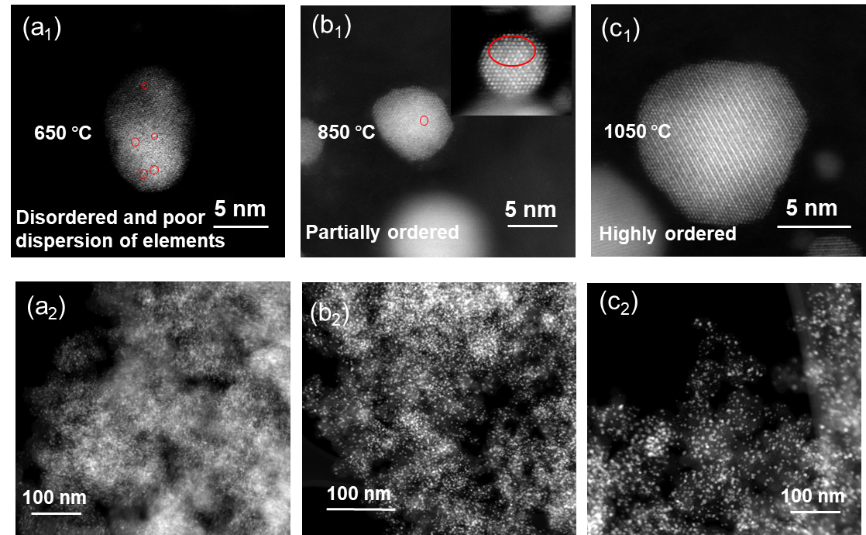


**Figure S5.** (a_1_-c_1_) HAADF-STEM image of the intermetallic PtIr-iHEA alloys obtained under 650°C, 850°C, and 1050°C; (a_2_-c_2_) The corresponding STEM images for the intermetallic PtIr-iHEA alloys

*Ex-situ* XRD and TEM analyses were conducted to investigate phase evolution at elevated temperatures. The high-entropy alloy undergoes significant structural transitions from a disordered solid solution to an ordered intermetallic alloy as the annealing temperature increases from 650 °C to 1050 °C (**Figure S4a**), confirmed by the intensifying superlattice diffraction peaks below 40° and the (111) peaks shifting to higher angles (**Figure S4b**), achieveing an ordering degree of ~90 % after annealing at 1050 °C(**Figure S4c**), revealing the temperature-dependent ordering behavior of the high entropy alloy. Additionally, HAADF-STEM images show non-uniform atomic distributions within the particles at 650 °C and 850 °C, indicating low alloy ordering degrees, while pyrolysis at 1050 °C results in a highly ordered atomic arrangement (**Figure S5a_1_-c_1_**), aligning with the XRD results.


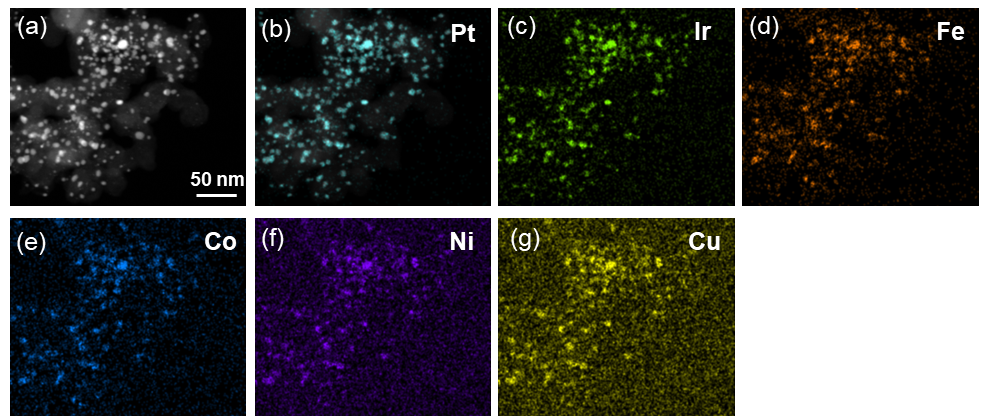


**Figure S6** (a) STEM image of the intermetallic PtIr-iHEA alloy; (b-g) The corresponding EDS mapping for Pt, Ir, Fe, Co, Ni, and Cu.


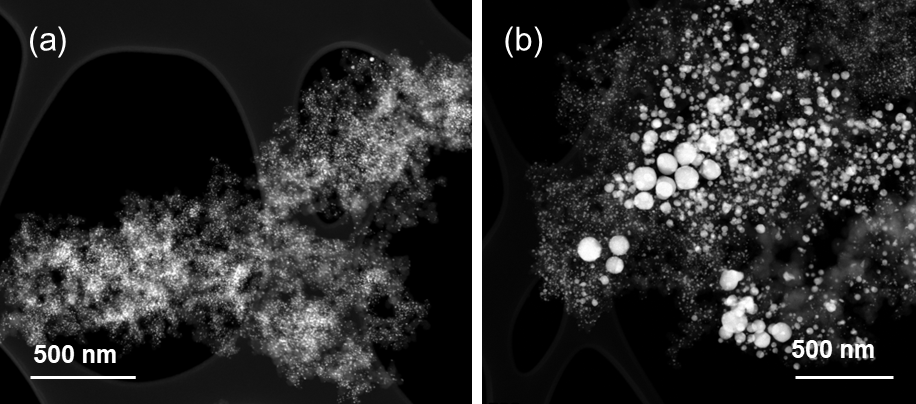


**Figure S7** (a-b) STEM images of PtIr-iHEA obtained at 1050°C with and without the addition of MSA.

**Table S1** Elemental composition of PtIr-iHEA alloy determined by ICP-MS

| Element | Mass ratio (wt%) |
| --- | --- |
| Pt | 18.69 |
| Ir | 4.15 |
| Fe | 1.30 |
| Co | 1.38 |
| Ni | 1.32 |
| Cu | 1.43 |

**Anti-sintering mechanism**


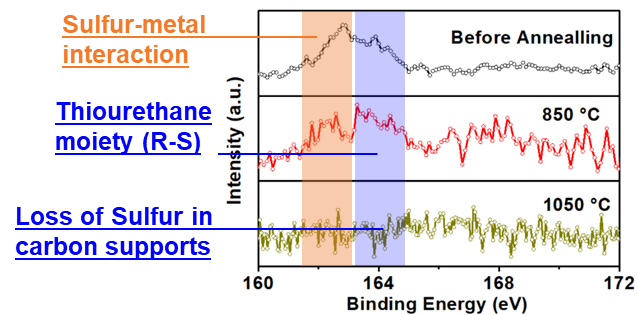


**Figure S8.** XPS spectra for PtIr-iHEA/C before annealing, annealed at 850°C and 1050°C


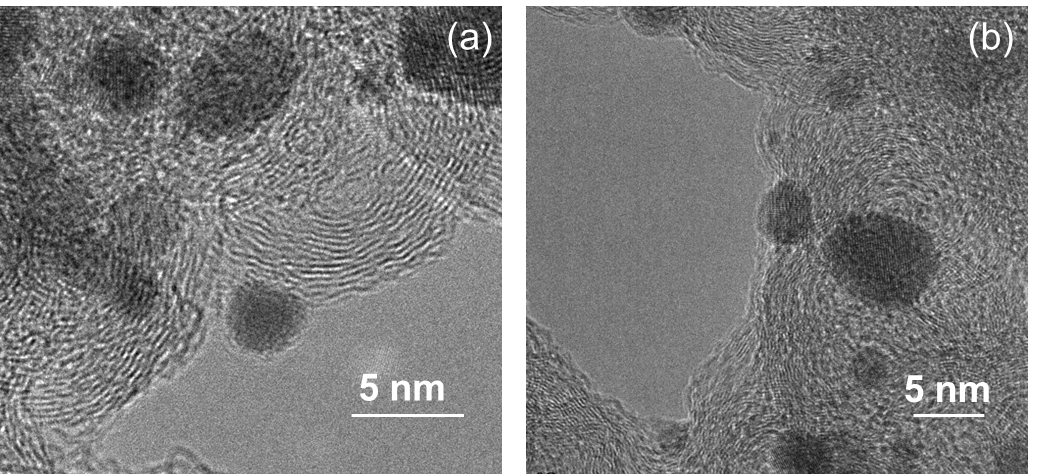


**Figure S9.** (a-b) TEM images of PtIr-iHEA/C catalyst (1050°C) and after air etching.

As shown in **Figure S7**, metal-sulfur interactions (~162 eV)^7^ in the XPS spectra of S *2p* during annealing provide evidence for the strong sintering resistance of PtIr-iHEA nanoparticles because of strong metal-S interaction. These interactions stabilize the nanoparticles by anchoring them onto the sulfur-doped carbon support (~164 eV, S-C configuration formation,^8, 9^ preventing temperature-dependent sintering. Furthermore, the weak metal-S peak in the alloy annealed at 1050°C suggests minimal sulfur residues in the final catalysts due to the high temperature.^10^ Instead, the nanoparticles are covered by a few-layer carbon, likely derived from the recrystallization of amorphous domains on the carbon supports, which can be removed after mild air etching. (**Figure S8**),^11^ further sterically hindering sintering**.**


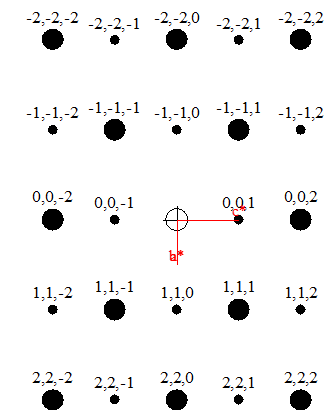


**Figure S10**. Simulated FFT patterns of PtIr-iHEA/C catalyst at zone axis of [1 -1 0]

12
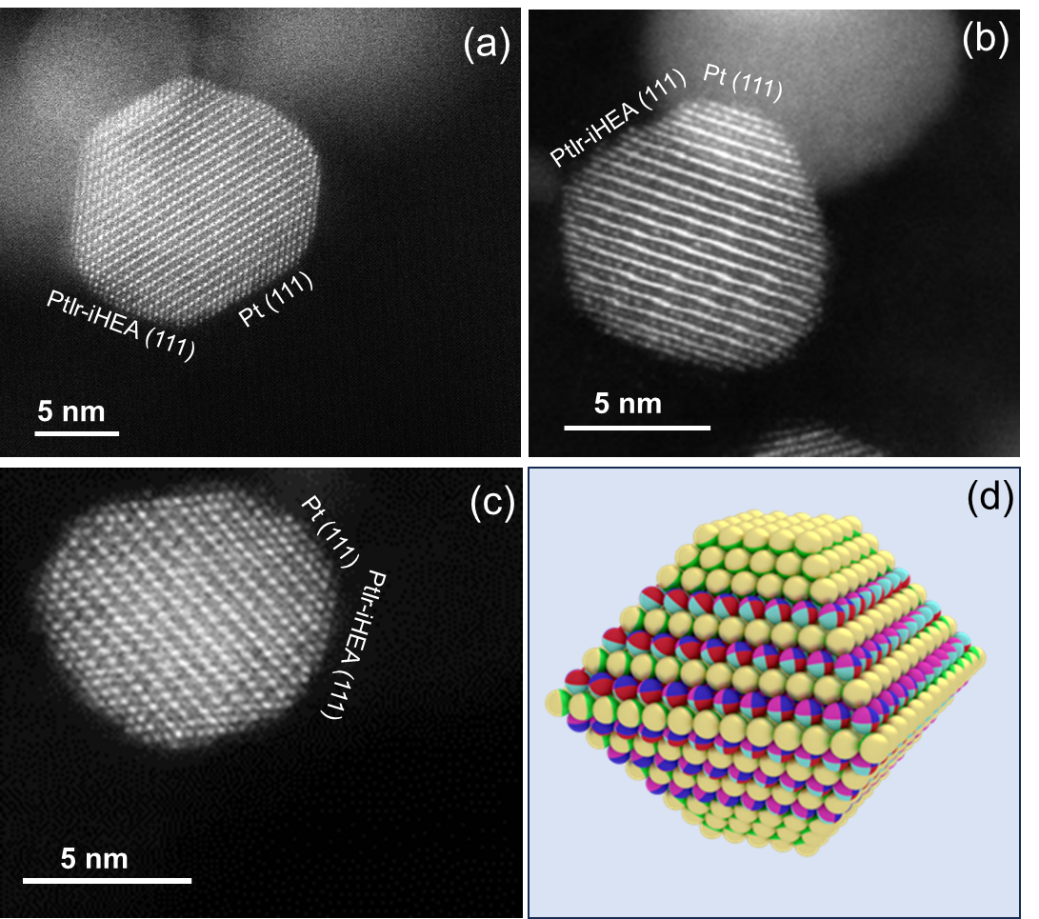


**Figure S11** Exposed active planes (a-c) HAADF-STEM images of PtIr-iHEA nanoparticles; (d) Illustration for the crystal orientation of the observed nanoparticles in (a-c).


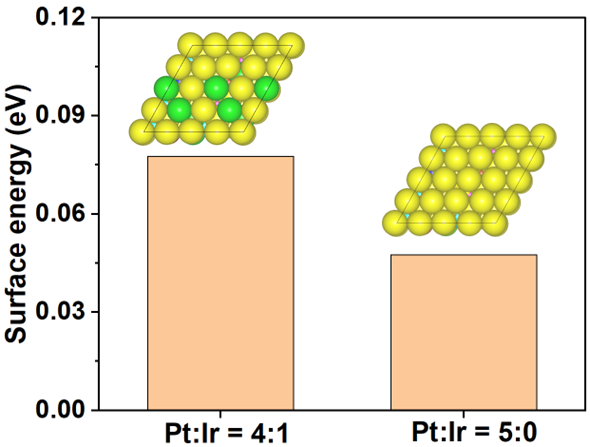


**Figure S12** Surface energies of noble metal shells with different ratios of Pt:Ir on PtIr-iHEA nanoparticles

The compositions of noble shells on PtIr-iHEA nanoparticles are determined by the surface energies of the shells with and without Ir doping. Pt atoms tend to segregate onto the surface of the alloy (Ir doping is beneficial for the durability of Pt shell.^12^ . Thereby, the noble shells are considered Pt shells for later discussions.


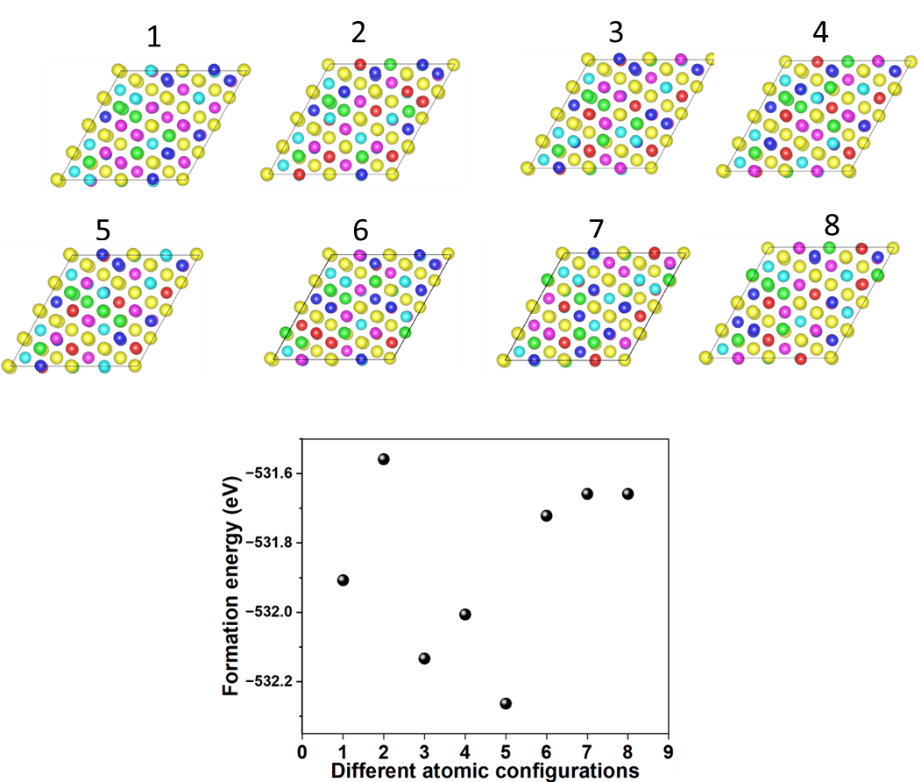


**Figure S13** Calculated formation energy for PtIr-iHEA with different atomic configurations, with the lowest being the most stable phase.

The formation energies for different atomic arrangements show very similar values between -531.62 eV and 532.23 eV, indicating the multi-possible atomic arrangements of the high entropy alloy. Therefore, it is reasonable to choose the most stable one as the model catalyst in the randomly picked configurations.





**Figure S14** XANES spectra of Ir L3 edge


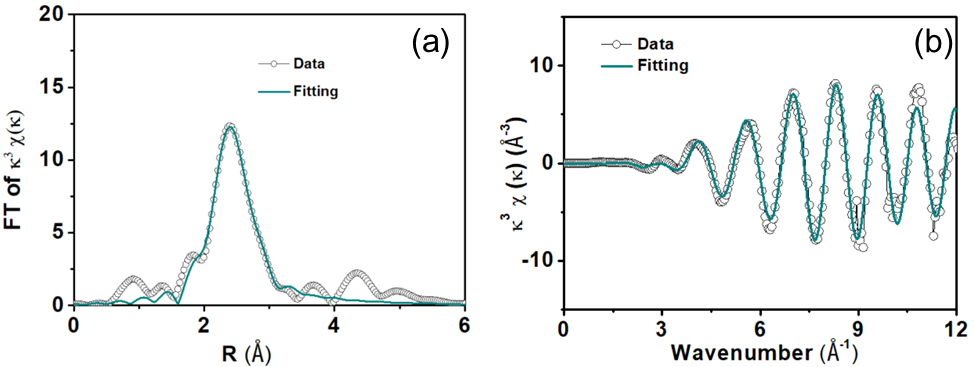


**Figure S15** Comparison of the experimental k3-weighted EXAFS and the fitting curves in the (a) R space and (b) K space for the as-prepared PtIr-iHEA catalyst at Pt L3-edge

**Table S2** Fitting parameters for PtIr-iHEA nanoparticles at Pt L3-edge

| Sample | Path | CN^[a]^ | R(Å)^[b]^ | σ^2^(×10^-3^ Å^2^ )^[c]^ | ΔE (eV)^[d]^ | R factor |
| --- | --- | --- | --- | --- | --- | --- |
| PtIr-iHEA/C | Pt-TM | 3.63±1.0 | 2.64±0.02 | 8.12 | 6.049 | 0.013 |
|  | Pt-Pt(Ir) | 4.64±0.75 | 2.70±0.01 | 3.78 |  |  |

[a] coordination numbers; [b] the internal atomic distance; [c] the edge-energy shift. [d]Debye-Waller factor;


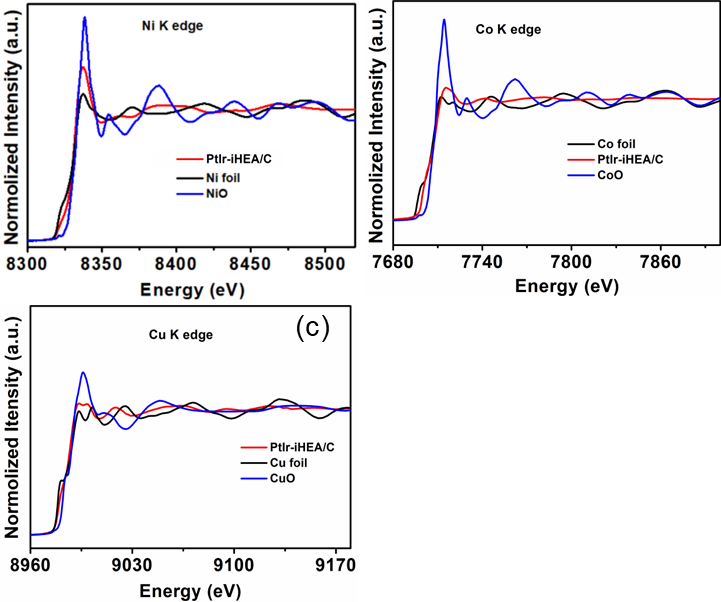


**Figure S16** (a-c) XANES spectra of Ni K edge, Co K edge, and Cu K edge, respectively


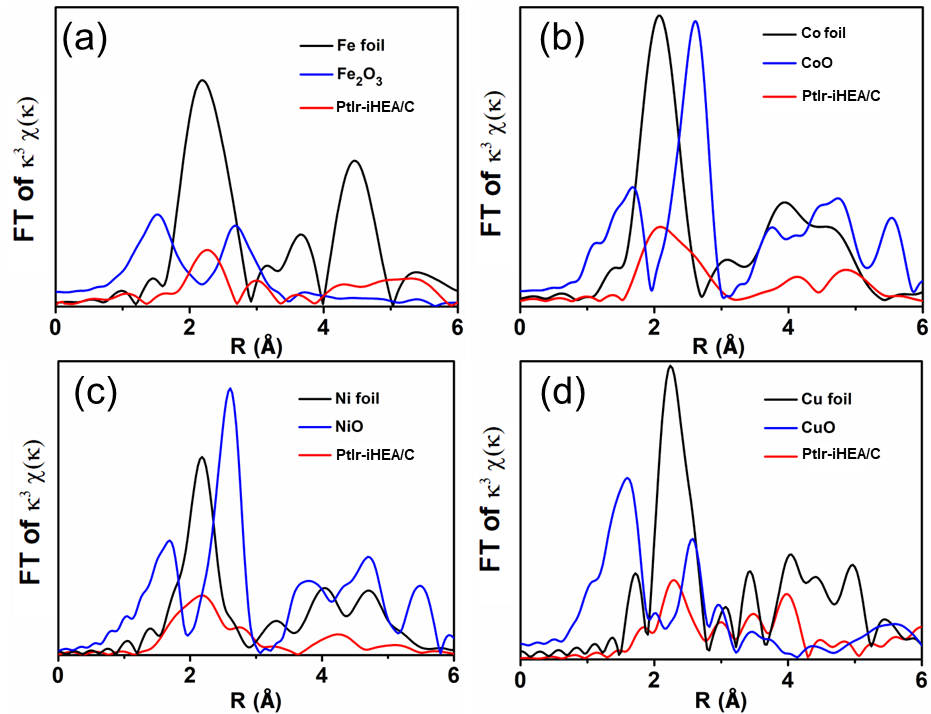


**Figure S17** Comparison of the k3-weighted EXAFS for elements in PtIr-iHEA/C and the standard samples in the R space at K edge. (a-d) Fe, Co, Ni, and Cu, respectively.





**Figure S18** XPS valence spectra for Pt element in Pt foil and PtIr-iHEA/C


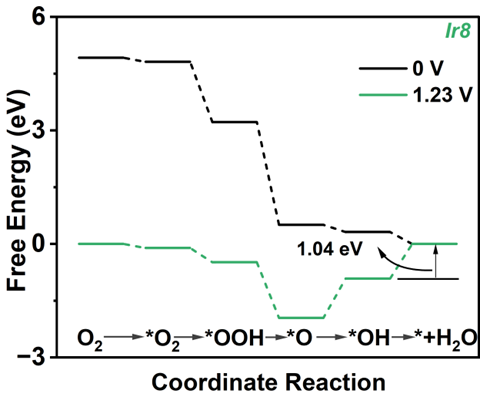


**Figure S19** Calculated free-energy diagram of ORR on Ir sites


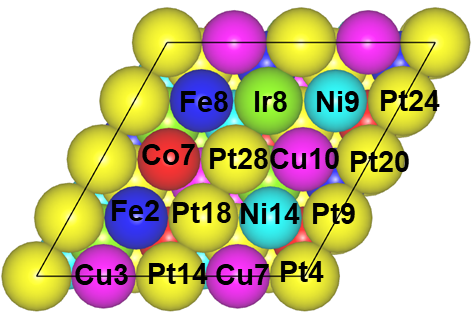


**Figure S20** The surface atomic compositions for the most stable PtIr-iHEA (111)


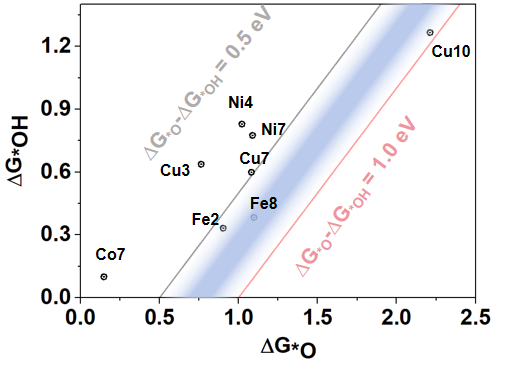


**Figure S21** Activity of each site on PtIr-iHEA (111)





**Figure S 22.** Tafel slopes for PtIr-iHEA/C, Pt/C, and PtIr-PiHEA/C


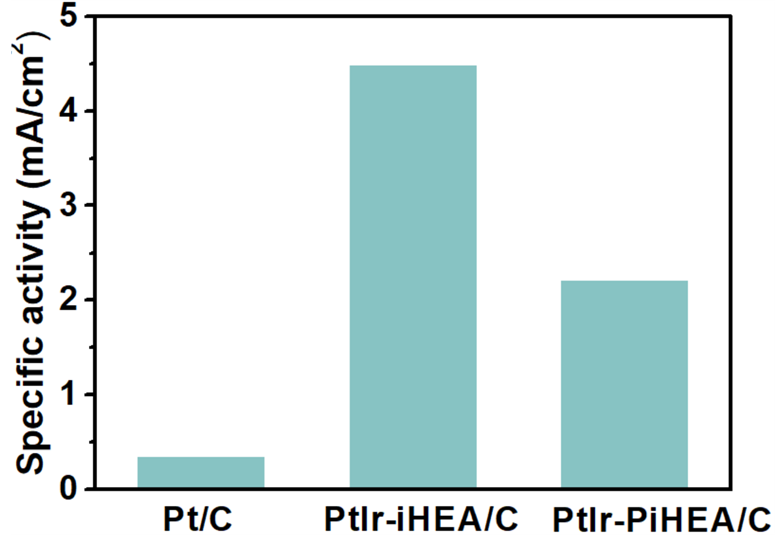


**Figure S23.** Specific activities for PtIr-iHEA/C, Pt/C, and PtIr-PiHEA/C


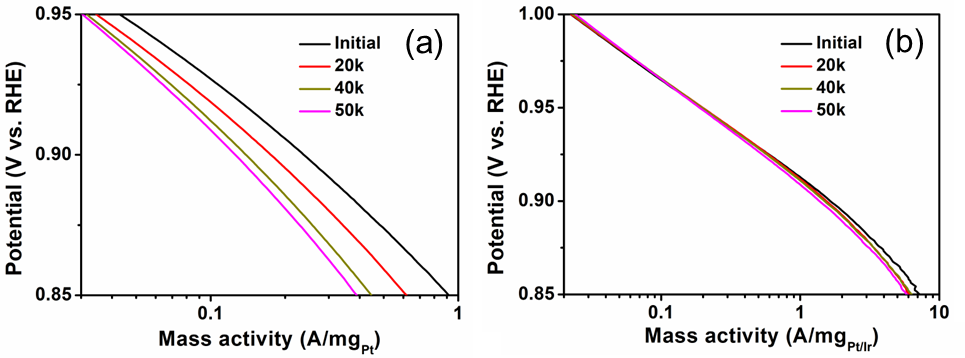


**Figure S24.**. (a-b) Mass activity curves of Pt/C, PtIr-iHEA/C with potential cycling


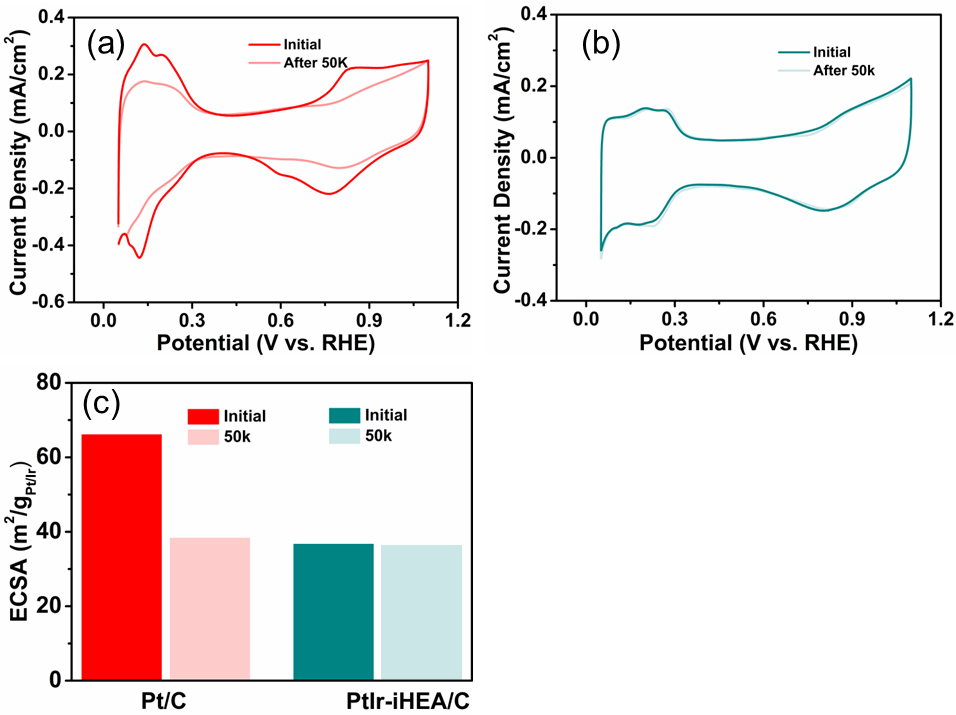


**Figure S25.** (a-b) CV curves for PtIr-iHEA/C and Pt/C catalysts before and after the durability test, respectively. (c) ECSA changed of PtIr-iHEA/C and Pt/C catalyst before and after the durability test.


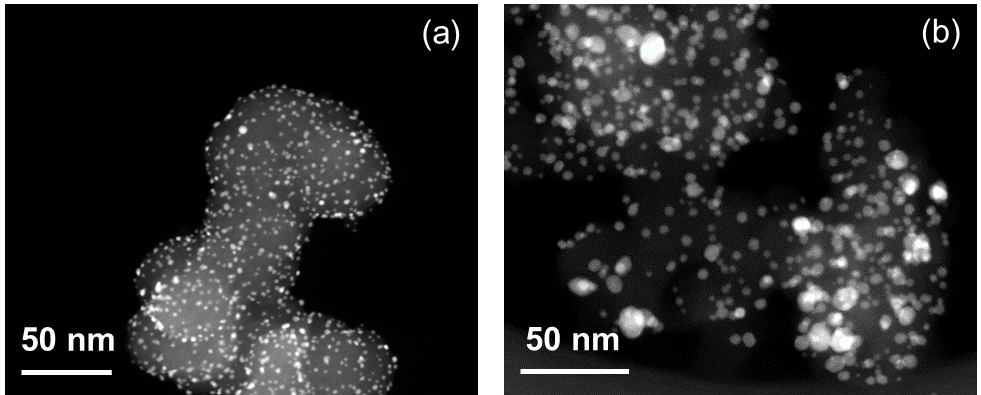


**Figure S26.** (a-b) STEM images of Pt/C before and after the durability test.


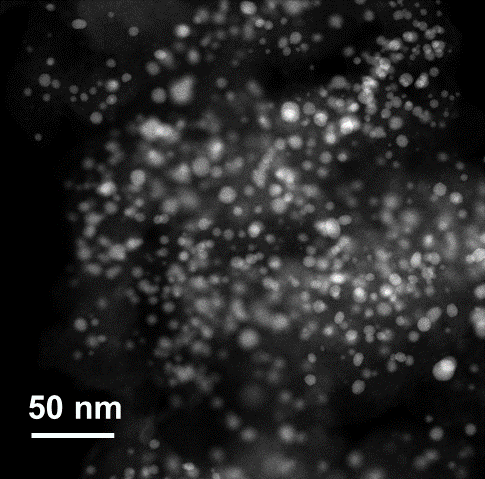


**Figure S27.** STEM image of PtIr-iHEA/C after the durability test


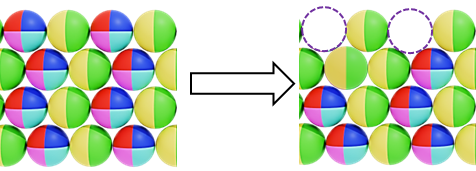


**Figure S28.** Schematic of PtIr-iHEA(111) evolution during activation process.


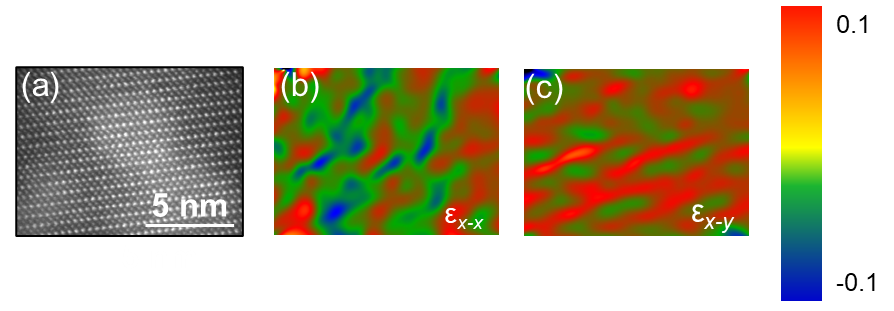


**Figure S29.** (a-b) HAADF-STEM image of PtIr-iHEA nanoparticles and the corresponding strain distributions within the nanoparticles (ε_x-x_, ε_x-y_), respectively


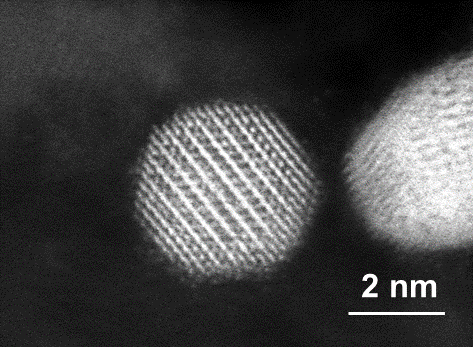


**Figure S30.** HAADF-STEM image of PtIr-iHEA@Pt nanoparticles


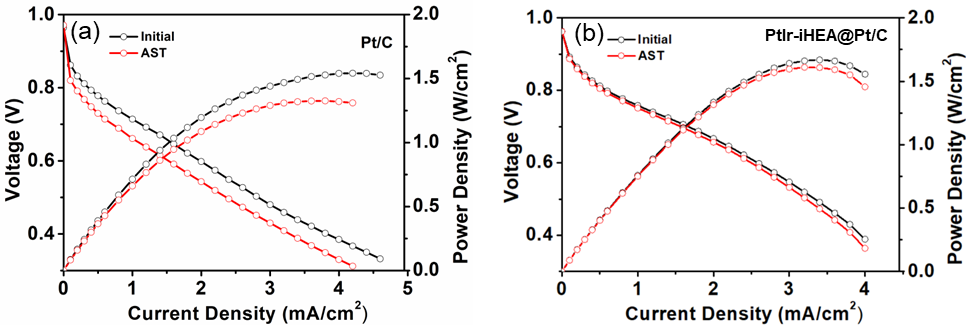


**Figure S31.** (a-b) MEA performances of Pt/C and the PtIr-iHEA @Pt/C catalyst under H_2_-O_2_, respectively





**Figure S32.** Voltage loss at 0.8 A/cm^2^ after AST for Pt/C and the PtIr-iHEA@Pt/C


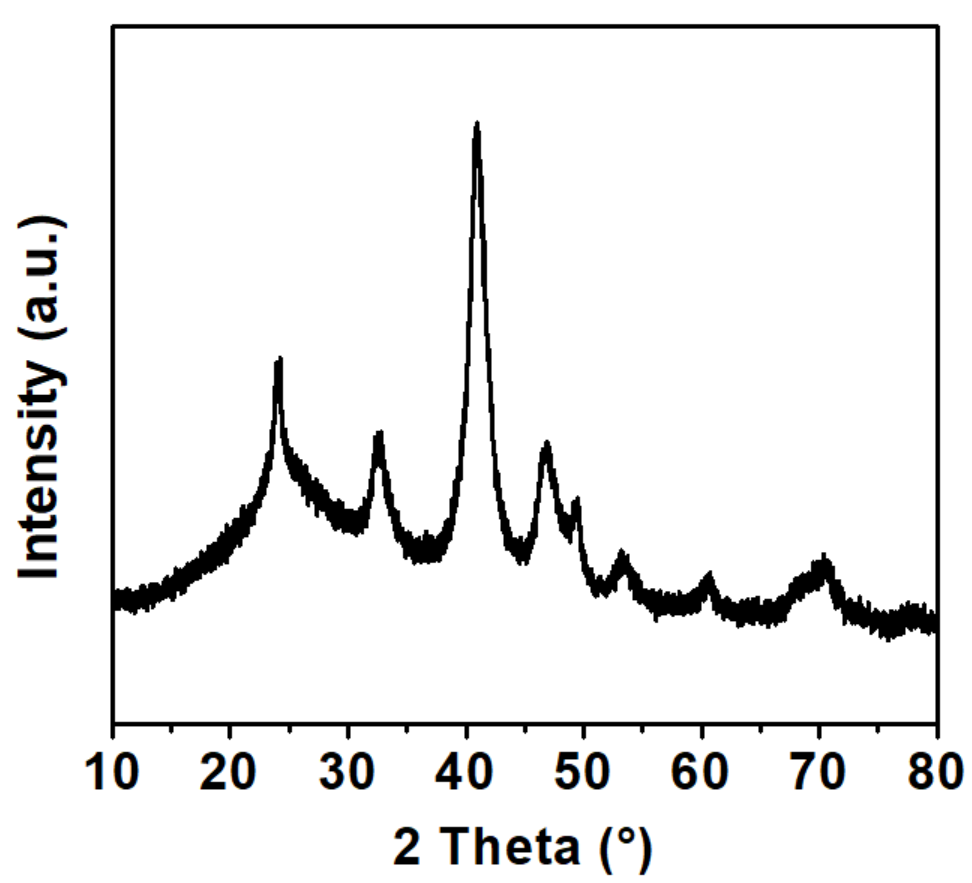


**Figure S33.** XRD pattern of the PtIr-iHEA@Pt/C catalyst after the durability test.

**Table S3.** Performance comparations between the as-studied catalyst and the reported catalysts in H_2_-air PEMFCs

| Sample | Peak power density  (W/cm^2^) | Voltage loss @ 0.8A/cm^2^  (mV) | MA retention  (at 0.9 V) | Normalized peak power density  (W/mg_Pt_) | Reference |
| --- | --- | --- | --- | --- | --- |
| i-CoPt@Pt/KB | 1.28 | 29 | 0.621 | 12.8 | 13 |
| PtIr-iHEA/C | 1.13 | 13 | 0.797 | 11.3 | *This work* |
| L10-Cr-PtFe/C | 1.3 | 10 | 0.751 | 10.4 | 14 |
| L1_0_-PtFe/C | 1.2 | 33 | 0.495 | 9.6 |  |
| PtNiMoAu | 0.92 | 25 | 0.774 | 9.2 | 15 |
| Gd−O−Pt_3_Ni | 1.04 | 19 | 0.660 | 10.4 | 16 |
| PtCo@Gng | 0.98 | 18.8 | 0.663 | 14 | 17 |
| STG-PtCo | 1.18 | 21 | 0.791 | 11.8 | 18 |
| PtCo/HSC-a | - | 21 | 0.464 | - | 19 |
| Pt_3_FeCo NSs/C | 0.24 | 40 | 0.672 | 1.3 | 20 |
| L10−Pt_2_CuGa/C | 1.21 | 28 | 0.653 | 12.1 | 21 |
| TKK-PtCo | - | 32 | 0.331 | - | 22 |
| PCNMC_Co8Zn7 | 0.93 | 20 | 0.707 | 9.3 | 23 |

Table S4 List of adsorption energies, zero-point energy, and entropy

| slab | intermediates | Eads (eV) | ZPE (eV) | S (eV/K) |
| --- | --- | --- | --- | --- |
| Pure  Pt (111) | Pt-OO | 4.383 | 0.141 | 0.000415898 |
|  | Pt-OOH | 3.974 | 0.413 | 0.000633909 |
|  | Pt-O | 1.846 | 0.062 | 0.000171055 |
|  | Pt-OH | 0.716 | 0.337 | 0.000456146 |
| Pt (111) layer on PtIr-iHEA(111) | Pt-OO | 4.597 | 0.143 | 0.000395774 |
|  | Pt-OOH | 3.892 | 0.435 | 0.000613785 |
|  | Pt-O | 2.245 | 0.058 | 0.000194533 |
|  | Pt-OH | 0.977 | 0.331 | 0.000315278 |
| Pt18  (PtIr-iHEA (111)) | Pt18-OO | 4.901 | 0.122 | 0.000456146 |
|  | Pt18-OOH | 3.989 | 0.420 | 0.000526581 |
|  | Pt18-O | 1.752 | 0.064 | 0.000160993 |
|  | Pt18-OH | 0.854 | 0.330 | 0.000298507 |
| Fe8  (PtIr-iHEA (111)) | Fe8-OO | 4.208 | 0.126 | 0.000452792 |
|  | Fe8-OOH | 3.433 | 0.395 | 0.000597015 |
|  | Fe8-O | 1.042 | 0.075 | 0.000140869 |
|  | Fe8-OH | 0.177 | 0.334 | 0.000395774 |
| Ir  (PtIr-iHEA (111)) | Ir-OO | 4.841 | 0.120 | 0.00049304 |
|  | Ir-OOH | 2.995 | 0.390 | 0.000553413 |
|  | Ir-O | 0.542 | 0.060 | 0.000315278 |
|  | Ir-OH | 0.126 | 0.332 | 0.000479624 |
| PtIr-iHEA (111) | Co7-O | 0.115 | 0.074 | 0.000140869 |
|  | Co7-OH | -0.176 | 0.353 | 0.000251551 |
|  | Cu3-O | 0.745 | 0.065 | 0.000157639 |
|  | Cu3-OH | 0.380 | 0.347 | 0.000305215 |
|  | Cu7-O | 1.048 | 0.072 | 0.000134161 |
|  | Cu7-OH | 0.339 | 0.326 | 0.000221365 |
|  | Cu10-O | 2.180 | 0.038 | 2.01241E-05 |
|  | Cu10-OH | 1.052 | 0.316 | 0.000338756 |
|  | Fe2-O | 0.962 | 0.056 | 0.000389066 |
|  | Fe2-OH | 0.114 | 0.315 | 0.00032534 |
|  | Fe8-O | 1.042 | 0.075 | 0.000140869 |
|  | Fe8-OH | 0.177 | 0.334 | 0.000395774 |
|  | Ni4-O | 0.913 | 0.147 | 0.000130807 |
|  | Ni4-OH | 0.829 | 0.323 | 0.001083347 |
|  | Ni7-O | 1.056 | 0.072 | 0.000134161 |
|  | Ni7-OH | 0.589 | 0.328 | 0.00047627 |

* Adsorption free energies for various oxygenated species are calculated with respect to H_2_O and H_2_ instead of X in the gas. For example, the adsorption free energy of O* (ΔGAO*) is calculated by the reaction free energy of *  +H_2_O ↔ *O  +  H_2_

* T=298.15 K

**Reference**

1. Kresse, G.; Joubert, D., From ultrasoft pseudopotentials to the projector augmented-wave method. *Physical Review B* **1999,** *59* (3), 1758-1775.

2. Perdew, J. P.; Burke, K.; Ernzerhof, M., Generalized gradient approximation made simple. *Physical review letters* **1996,** *77* (18), 3865.

3. Wang, V.; Xu, N.; Liu, J.-C.; Tang, G.; Geng, W.-T., VASPKIT: A user-friendly interface facilitating high-throughput computing and analysis using VASP code. *Computer Physics Communications* **2021,** *267*, 108033.

4. Nørskov, J. K.; Rossmeisl, J.; Logadottir, A.; Lindqvist, L.; Kitchin, J. R.; Bligaard, T.; Jonsson, H., Origin of the overpotential for oxygen reduction at a fuel-cell cathode. *The Journal of Physical Chemistry B* **2004,** *108* (46), 17886-17892.

5. Strzelec, K.; Bączek, N., Platinum complex catalysts immobilized on epoxy resins cured with polythiourethane hardeners. *Open Chemistry* **2015,** *13* (1).

6. Atzei, D.; De Filippo, D.; Rossi, A.; Porcelli, M., X-ray photoelectron spectra of Pd(II) and Pt(II) complexes with 1,3-thiazolidine-2-thione. A quantum mechanics study on the free ligand. *Spectrochimica Acta Part A: Molecular and Biomolecular Spectroscopy* **2001,** *57* (5), 1073-1083.

7. Lin, B.; Zhou, Y.; Xu, B.; Zhu, C.; Tang, W.; Niu, Y.; Di, J.; Song, P.; Zhou, J.; Luo, X.; Kang, L.; Duan, R.; Fu, Q.; Liu, H.; Jin, R.; Xue, C.; Chen, Q.; Yang, G.; Varga, K.; Xu, Q.; Li, Y.; Liu, Z.; Liu, F., 2D PtS nanorectangles/g-C3N4 nanosheets with a metal sulfide–support interaction effect for high-efficiency photocatalytic H2 evolution. *Materials Horizons* **2021,** *8* (2), 612-618.

8. Yang, C.-L.; Wang, L.-N.; Yin, P.; Liu, J.; Chen, M.-X.; Yan, Q.-Q.; Wang, Z.-S.; Xu, S.-L.; Chu, S.-Q.; Cui, C., Sulfur-anchoring synthesis of platinum intermetallic nanoparticle catalysts for fuel cells. *Science* **2021,** *374* (6566), 459-464.

9. Wang, Y.; Gong, N.; Liu, H.; Ma, W.; Hippalgaonkar, K.; Liu, Z.; Huang, Y., Ordering‐Dependent Hydrogen Evolution and Oxygen Reduction Electrocatalysis of High‐Entropy Intermetallic Pt4FeCoCuNi. *Advanced Materials* **2023,** *35* (28), 2302067.

10. Garsany, Y.; Baturina, O. A.; Swider-Lyons, K. E., Oxygen Reduction Reaction Kinetics of SO2-Contaminated Pt3Co and Pt/Vulcan Carbon Electrocatalysts. *Journal of The Electrochemical Society* **2009,** *156* (7), B848.

11. Natarajan, S. K.; Cossement, D.; Hamelin, J., Synthesis and Characterization of Carbon Nanostructures as Catalyst Support for PEMFCs. *Journal of The Electrochemical Society* **2007,** *154* (3), B310.

12. Ma, Y.; Balbuena, P. B., Role of iridium in Pt-based alloy catalysts for the ORR: Surface adsorption and stabilization studies. *Journal of the Electrochemical Society* **2010,** *157* (6), B959.

13. Yoo, T. Y.; Lee, J.; Kim, S.; Her, M.; Kim, S.-Y.; Lee, Y.-H.; Shin, H.; Jeong, H.; Sinha, A. K.; Cho, S.-P.; Sung, Y.-E.; Hyeon, T., Scalable production of an intermetallic Pt–Co electrocatalyst for high-power proton-exchange-membrane fuel cells. *Energy & Environmental Science* **2023,** *16* (3), 1146-1154.

14. Liu, X.; Wang, Y.; Liang, J.; Li, S.; Zhang, S.; Su, D.; Cai, Z.; Huang, Y.; Elbaz, L.; Li, Q., Introducing Electron Buffers into Intermetallic Pt Alloys against Surface Polarization for High-Performing Fuel Cells. *Journal of the American Chemical Society* **2024,** *146* (3), 2033-2042.

15. Gao, L.; Sun, T.; Chen, X.; Yang, Z.; Li, M.; Lai, W.; Zhang, W.; Yuan, Q.; Huang, H., Identifying the distinct roles of dual dopants in stabilizing the platinum-nickel nanowire catalyst for durable fuel cell. *Nature Communications* **2024,** *15* (1), 508.

16. Yang, L.; Bai, J.; Zhang, N.; Jiang, Z.; Wang, Y.; Xiao, M.; Liu, C.; Zhu, S.; Xu, Z. J.; Ge, J.; Xing, W., Rare Earth Evoked Subsurface Oxygen Species in Platinum Alloy Catalysts Enable Durable Fuel Cells. *Angewandte Chemie International Edition* **2024,** *63* (7), e202315119.

17. Zhao, Z.; Liu, Z.; Zhang, A.; Yan, X.; Xue, W.; Peng, B.; Xin, H. L.; Pan, X.; Duan, X.; Huang, Y., Graphene-nanopocket-encaged PtCo nanocatalysts for highly durable fuel cell operation under demanding ultralow-Pt-loading conditions. *Nature Nanotechnology* **2022,** *17* (9), 968-975.

18. Song, T.-W.; Xu, C.; Sheng, Z.-T.; Yan, H.-K.; Tong, L.; Liu, J.; Zeng, W.-J.; Zuo, L.-J.; Yin, P.; Zuo, M.; Chu, S.-Q.; Chen, P.; Liang, H.-W., Small molecule-assisted synthesis of carbon supported platinum intermetallic fuel cell catalysts. *Nature Communications* **2022,** *13-1* (1), 6521.

19. Ramaswamy, N.; Kumaraguru, S.; Gu, W.; Kukreja, R. S.; Yu, K.; Groom, D.; Ferreira, P., High-Current Density Durability of Pt/C and PtCo/C Catalysts at Similar Particle Sizes in PEMFCs. *Journal of The Electrochemical Society* **2021,** *168* (2), 024519.

20. Bu, L.; Liang, J.; Ning, F.; Huang, J.; Huang, B.; Sun, M.; Zhan, C.; Ma, Y.; Zhou, X.; Li, Q.; Huang, X., Low-Coordination Trimetallic PtFeCo Nanosaws for Practical Fuel Cells. *Advanced Materials* **2023,** *35* (11), 2208672.

21. Liu, X.; Zhao, Z.; Liang, J.; Li, S.; Lu, G.; Priest, C.; Wang, T.; Han, J.; Wu, G.; Wang, X.; Huang, Y.; Li, Q., Inducing Covalent Atomic Interaction in Intermetallic Pt Alloy Nanocatalysts for High-Performance Fuel Cells. *Angewandte Chemie International Edition* **2023,** *62* (23), e202302134.

22. Li, S.; Li, J.-J.; Xu, C.; Zhang, L.; Li, A.; Song, T.-W.; Zhang, W.; Tong, L.; Liang, H.-W., Multigram-Scale Synthesis of High-Pt-Content PtCo Intermetallic Catalysts for Proton Exchange Membrane Fuel Cells. *ACS Materials Letters* **2024,** *6* (2), 706-712.

23. Chen, Z.; Hao, C.; Yan, B.; Chen, Q.; Feng, H.; Mao, X.; Cen, J.; Tian, Z. Q.; Tsiakaras, P.; Shen, P. K., ZIF-Mg(OH)2 Dual Template Assisted Self-Confinement of Small PtCo NPs as Promising Oxygen Reduction Reaction in PEM Fuel Cell. *Advanced Energy Materials* **2022,** *12* (32), 2201600.
